# Interaction of lncRNA LENT with DHX36 regulates translation and suppresses autophagy in melanoma

**DOI:** 10.1038/s41419-025-08296-3

**Published:** 2025-12-19

**Authors:** Alexandre Haller, Giovanni Gambi, Mattia D’Agostino, Guillaume Davidson, Antonin Lallement, Gabrielle Mengus, Chadia Nahy, Nadia Messaddeq, Guillaume Bec, Angelita Simonetti, Eric Ennifar, Irwin Davidson

**Affiliations:** 1 Institut de Génétique et de Biologie Moléculaire et Cellulaire, Illkirch, France; 2 Centre National de la Recherche Scientifique, UMR7104, Illkirch, France; 3 Institut National de la Santé et de la Recherche Médicale, U1258, Illkirch, France; 4 Université de Strasbourg, Illkirch, France; 5 NYU Grossman School of Medicine, Langone Medical Center, New York, NY USA; 6 Institut de Biologie Moléculaire et Cellulaire du CNRS, Strasbourg, France; 7 Equipe Labélisée Ligue contre le Cancer, Strasbourg, France

**Keywords:** Melanoma, Long non-coding RNAs, Macroautophagy

## Abstract

The melanocyte lineage-determining Microphthalmia-associated transcription factor (MITF) drives proliferation and survival of melanocytic melanoma cells through regulation of both coding genes and long non-coding RNAs (LncRNAs). Here we characterize LINC00520 (hereafter called LncRNA ENhancer of Translation, LENT) regulated by MITF and strongly expressed in melanocytic melanoma cells. LENT is essential for the proliferation and survival of cultured melanocytic melanoma cells and xenograft tumors. LENT interacts with the G4 quadruplex resolvase DHX36, and both associate with the ribosome in the 80S and light polysome fractions. LENT modulates DHX36 association with a collection of mRNAs regulating their engagement with polysomes and fine-tuning their subsequent translation. These mRNAs encode proteins involved in endoplasmic reticulum (ER) and mitochondrial homeostasis as well as autophagy. Consequently, LENT silencing leads to extensive autophagy and mitophagy, compromised oxidative metabolic capacity, accompanied by an accumulation and mis-localization of mitochondrial proteins leading to proteotoxic stress and apoptosis. The LENT-DHX36 axis therefore fine-tunes translation of proteins involved in ER and mitochondrial homeostasis, suppressing autophagy and promoting survival and proliferation of melanoma cells.

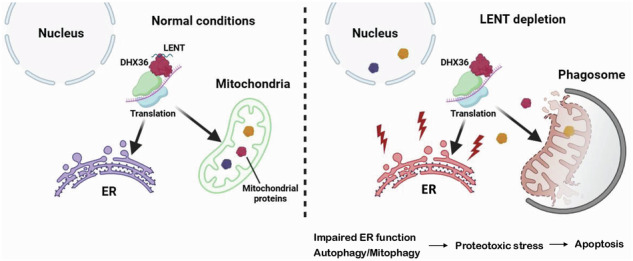

## Introduction

Melanoma tumors are notoriously heterogeneous, with melanoma cells adopting multiple cell states with differing proliferative, invasive and stem cell capacities [1–7]. Intra-tumor heterogeneity is a major determinant of therapeutic resistance, with mesenchymal-type cells playing a critical role in targeted and immune checkpoint resistance [4, 7]. The transcription programs associated with the different cell states are driven by a host of transcription factors, with the lineage-defining MITF (Microphthalmia-associated transcription factor) and SOX10 driving the more differentiated melanocytic cell state, while AP1, TEAD, PRRX1 and TCF4 drive the undifferentiated mesenchymal state [6–11]. Other intermediate states have been defined, such as the neural crest stem cell (NCSC)-like state that plays a key role in minimal residual disease and the emergence of drug-resistant populations [3, 12].

In melanocytic melanoma cells, MITF and SOX10 bind together at cis-regulatory elements to promote expression of genes driving proliferation, survival and oxidative metabolism [8, 9]. While these factors regulate multiple coding genes, they also regulate the expression of long non-coding (lnc)RNAs, such as the melanoma-specific lncRNA SAMMSON (LINC01212), essential for melanoma cell proliferation and survival through coordinating mitochondrial and cytoplasmic translation [13, 14]. SAMMSON silencing induced mitochondrial precursor overaccumulation stress (mPOS), a form of proteotoxic stress, resulting in melanoma cell death. SOX10 also regulates the melanoma-specific lncRNA LENOX (LINC00518) that interacts with the small GTPase RAP2C, promoting its interaction with DRP1 and impairing mitochondrial fission through enhanced DRP1 S637 phosphorylation [15]. LENOX potentiates oxidative phosphorylation metabolism to promote melanoma cell survival and resistance to MAP kinase inhibitors [16].

Here we characterize LINC00520 (hereafter called LncRNA ENhancer of Translation, LENT), strongly expressed in melanocytic melanoma cells under the regulation of MITF and essential for proliferation and survival in vitro and in vivo. LENT interacts with the G4 quadruplex resolvase DHX36, and both associate with the 80S and light polysome fractions. LENT modulates DHX36 association with a collection of mRNAs regulating their engagement in polysomes and their subsequent translation. LENT coordinately regulates engagement with the light polysomes of mRNAs encoding proteins enriched in endoplasmic reticulum (ER) and lysosome homeostasis, as well as autophagy and mitophagy. Consequently, LENT silencing leads to extensive autophagy/mitophagy, compromised OxPhos capacity and accumulation and mis-localization of mitochondrial proteins, leading to proteotoxic stress and apoptosis. Our results are consistent with a model where LENT fine-tunes translation of proteins involved in ER, lysosomal and mitochondrial homeostasis by modulating the ability of ribosome-associated DHX36 to unwind G4 structures in their mRNAs and their engagement with polysomes. LENT, LENOX and SAMMSON hence constitute a set of melanoma-expressed lncRNAs that act coordinately to fine-tune translation and/or mitochondrial activity and promote melanoma cell proliferation and survival.

## Materials and methods

### Analysis of the TCGA-SKCM cohort

For analysis of TCGA-SKCM, raw-counts were retrieved, and primary tumors were separated from distant metastasis samples. The raw-counts matrices were normalized by sequencing depth using DESeq2 size-factors, and then gene-counts were divided by median transcript length. Consensus clustering was done in R using the ConsensusClusterPlus v3.17 package following the standard procedure. In short, matrices were filtered to keep only coding genes based on their biotype annotation, and the 5000 most variable genes were selected with the mad() function. The matrices were median-centered with sweep(), apply() and median() functions before performing consensus clustering with ConsensusClusterPlus() using base parameters. The number of clusters was selected based on the curve of the cumulative distribution function in order to define 4 clusters for primary tumors (CCP1-CCP4) and 5 clusters for distant metastasis samples (CCM1-CCM5).

### Cell culture and transfections

Melanoma cell lines SK-MEL-25, SK-MEL-25R (gifts from Dr L. Larue), SK-MEL-28, and 501mel (ATCC) were grown in RPMI1640 w/o HEPES medium supplemented with 10% fetal calf serum (FCS) and gentamycin (40 µg/mL); IGR-37 and IGR-39 in RPMI1640 w/o HEPES medium supplemented with 15% FCS and gentamycin (40 µg/mL). MM011, MM117, MM047, and MM099 (gifts from Dr J-C. Marine) were grown in HAM-F10 medium supplemented with 10% FCS, 5.2 mM glutamax, 25 mM Hepes, and penicillin/streptomycin (7.5 μg/mL). M229, M229R, M249, and M249R (gifts from Dr J-C. Marine) were grown in DMEM medium supplemented with glucose (4.5 g/L), 5% FCS, and penicillin/streptomycin (7.5 μg/mL). A375 cells were grown in DMEM medium supplemented with glucose (4.5 g/L), 10% FCS, and gentamycin (40 µg/mL). HEK293T cells were grown in DMEM medium supplemented with glucose (1 g/L), 10% FCS, and penicillin/streptomycin (7.5 μg/mL). HeLa cells were grown in DMEM medium with glucose (1 g/L), 5% FCS and gentamycin (40 µg/mL). To assess cell growth and viability, cells were stained with Trypan Blue (Invitrogen). Trametinib (GSK1120212) and dabrafenib (GSK2118436) were purchased from Selleckchem. SK-MEL-25, SK-MEL-28, A375, and 501mel were obtained from ATCC; all other cell lines were gifts from collaborators. All cell lines were regularly tested using the Venor GeM Mycoplasma Detection Kit, and used at less than 10 passages.

ASO and siRNA were transfected using Lipofectamine RNAiMAX (Invitrogen) with 20 nM of ASO (Qiagen) or siRNA (ThermoFisher Scientific). ASO and siRNA sequences are listed below. For ASO combination experiments, cells were transfected with 15 nM of LENT ASO and/or 15 nM of LENOX ASO and/or 5 nM of SAMMSON ASO. For trametinib+dabrafenib-GapmeR cotreatment, cells were cultured for 3 days in the presence or absence of Dabrafenib (100 nM) + Trametinib (100 nM), transfected with 15 nM of GapmeR and then cultured for an additional 3 days before harvesting. Colony-forming ability was assessed by plating 500 cells/9.6 cm^2^, waiting for 10 days, fixing cells in formalin and staining with 0.05% Crystal Violet solution (Sigma Aldrich).

### CRISPR interference

501mel cells were co-transfected with a plasmid expressing dead Cas9 protein fused to the Kruppel-associated box (KRAB) domain-containing KAP1 (dCas9-KAP1) and the red fluorescent protein mScarlet (pX-dCas9-KRAB-Scarlet), together with another plasmid expressing GFP and three single guide RNAs targeting the transcription start site of LENT (pcDNA3-sgRNA-GFP) or a control plasmid expressing GFP only (pCMV-GFP). Double Scarlet-GFP positive cells were sorted 24 h after co-transfection, stained with Cell Trace Violet and cultured for an additional 96 h.

### Plasmid cloning and lentiviral transduction

For the ectopic expression experiment, LENT cDNA was cloned into the pCW57-GFP-P2A-MCS vector (a gift from Adam Karpf; Addgene plasmid #71783; http://n2t.net/addgene:71783; RRID: Addgene_71783). LENT shRNA (shLENT) or a scrambled control (shCTRL) were cloned in LT3GEPIR (a gift from Johannes Zuber; Addgene plasmid #111177; http://n2t.net/addgene:111177; RRID: Addgene_111177). Lentiviral particles were produced in HEK293T cells, purified by ultracentrifugation, and resuspended in PBS. Lentiviruses were titrated with flow cytometry by measuring the GFP signal intensity in HEK293T infected with different dilutions of viruses. Melanoma cells were eventually infected at a multiplicity of infection (MOI) of 1 and selected by puromycin addition to the media (1 mg/mL) in every following passage.

### RNAscope

LENT and MITF RNAs were detected with the RNAscope assay (Advanced Cell Diagnostics, ACD) according to the manufacturer’s protocol. Patient sections were deparaffinized, incubated with hydrogen peroxide at room temperature for 10 min, boiled with target retrieval reagent for 15 min, and then treated with protease plus reagent at 40 °C for 30 min. Sections were hybridized with Hs-MITF probe (ACD, catalog no. 310951) and hs-LENT at 40 °C for 2 h. Probes for Hs-LENT were custom-designed by ACD. Hybridization signals were amplified and visualized with RNAscope Multiplex Fluorescent Reagent Kit v2 (ACD, catalog no. 323100). For co-detection of DHX36 with LENT, cells were fixed for 30 min with formaldehyde 3.7%, washed with PBS and incubated 10 min at room temperature with H_2_O_2_. After one wash in distilled water, primary antibody for DHX36 diluted in co-detection diluent (1/200) was added o/n at 4 °C. Slides were washed in PBS + tween 0.1% (PBST), fixed in formaldehyde 3.7% for 30 min, and washed again in PBST. Slides were treated with protease III and washed with PBS. LENT hybridization signals were amplified following the Multiplex Fluorescent Kit. Finally, DHX36 signal was developed by secondary antibody incubation (diluted 1/2000 in co-detection diluent), followed by tyramide signal amplification (TSA Plus Kit, NEL760001KT, Perkin Elmer). Images were captured with a confocal (Leica DMI6000) microscope. Mander’s and Pearson’s coefficients were calculated with the Fiji software using the JACoP plugin.

### Analysis of oxygen consumption rate in living cells

Oxygen consumption rate (OCR) was measured in an XF96 extracellular analyzer (Seahorse Bioscience). 20,000 transfected cells per well were seeded 48 h prior to the experiment. The cells were incubated at 37 °C and the medium was changed to XF base medium supplemented with 1 mM pyruvate, 2 mM glutamine, and 10 mM glucose for 1 h before OCR profiling with the Mitostress Test Kit sequentially exposed to 2 μM oligomycin, 1 μM carbonyl cyanide-p-trifluoromethoxyphenylhydrazone (FCCP), and 0.5 μM rotenone and antimycin A. Cells were washed with PBS, fixed with 3% PFA and permeabilized with 0.2% triton. Nuclei were counterstained with DAPI (1:500), and the number of cells per well was determined with a Celomics Cell Insight CX7 (ThermoFisher Scientific). Non-mitochondrial oxygen consumption was subtracted from the total values to designate true mitochondrial oxygen consumption values.

### Flow cytometry

To assess cell viability and proliferation, cells were stained with Cell Trace Violet (Invitrogen) on the day of transfection, harvested after 72 h and stained with Annexin V (BioLegend) and TOPRO-3 (Invitrogen) or the active caspase-3 Kit (BD Biosciences). Cell Trace Violet staining was performed following the manufacturer’s instructions and as previously described [15, 17]. All analyses were performed on an LSRII Fortessa (BD Biosciences), and data were analyzed with FlowJo software (TreeStar).

To analyze intracellular ROS, cells were stained in adherent conditions with CellRox Deep Red (ThermoFisher Scientific) at a final concentration of 500 nM following manufacturer instructions. After harvesting, cells were stained for active caspase-3 (BD Biosciences) and analyzed on a LSRII Fortessa (BD Biosciences). To induce reactive oxygen species (ROS), cells were treated with THBP (200 μM) for 30 min. To induce apoptosis, cells were treated with staurosporine (500 nM) for 16 h.

### LENT pulldown and LC/MS-MS analysis

501mel cells were grown in 15 cm petri dishes, harvested by trypsinization, washed, pelleted, resuspended in lysis buffer (Tris-HCl 20 mM pH 8, NaCl 200 mM, MgCl_2_ 2.5 mM, Triton 0.05%, DEPC water) supplemented with fresh DTT (1 mM), protease and phosphatase inhibitor cocktail (ThermoFisher Scientific) and RNAsin (ThermoFisher Scientific) and kept 20 min on ice. For crosslinked pulldown, petri dishes were exposed to 400 mJ/cm^2^ of UV radiation with a CL-1000 crosslinker (254 nm lamp), and the concentration of NaCl in lysis buffer was adjusted to 300 mM. Membranes were pelleted at 3000×*g* for 3 min at 4 °C and supernatant precleared for 1 h at 4 °C with 100 µg of streptavidin-coated sepharose beads (Cytiva). The lysate was incubated for 2 h with streptavidin-coated beads and 400 pmol anti-PCA3 or LENT-specific DNA biotinylated oligonucleotides (listed below). Beads were pelleted for 3 min at 3000×*g* and washed five times with lysis buffer. After the final wash, beads were divided for RNA and protein extraction. RNA was purified by TRI Reagent and isopropanol precipitation, digested with DNAse, reverse transcribed and analyzed by qPCR. Proteins were eluted by boiling beads in Laemmli sample buffer and separated on NuPAGE Novex 4% to 12% gradient gels. For mass spectrometry analysis, three independent experiments were performed, and the entire lane was excised after staining with Simply blue safe stain solution (Invitrogen). Analysis was performed at the Harvard Medical School Taplin Mass Spectrometry Facility, as described previously [9, 15].

### IGR37 xenograft model and ASO treatment

Swiss nude mice were purchased from Charles River Laboratories (France) and housed under specific pathogen-free conditions. Animal care, use, and experimental procedures were conducted in accordance with recommendations of the European Community (86/609/EEC), European Union (2010/63/UE) and the French National Committee (87/848). The ethics committee of IGBCM, in compliance with institutional guidelines, approved animal care and use (APAFIS#2023010611181767). Six-week-old male mice were injected on the rear flank with 3 × 10^6^ IGR37 cells resuspended in 100 µL of 1x PBS + Cultrex Basement Membrane Extract (ref. 3432–005–01; R&D Systems) with a 1:1 ratio. Tumor growth was monitored by caliper measurement every 2 days, and volume was calculated with the formula: (4/3 π) * (length/2) * (width/2) * (height/2). After tumors reached 100 mm^3^, mice were randomized and injected subcutaneously around 1 cm from the tumor every 2 days with 15 mg/kg of ASO for the LENT group, or not injected for the control group. After 2 weeks of treatment, mice were sacrificed, and primary tumors were dissected and mechanically lysed in TRI Reagent for RNA extraction or LSDB for protein extraction. No blinding was used in the CDX experiments.

### Immunofluorescence of fixed and live cells

Cells grown on Millicell EZ slides (Millipore) were fixed with 4% paraformaldehyde for 15 min. After two washes with PBS buffer, they were permeabilized in PBS + Triton X-100 0.1% for 5 min and blocked with PBS + 10% FCS for 20 min. Primary antibodies were incubated overnight at 4 °C, and after three washes with PBS + Triton 0.1%, cells were stained for 1 h at room temperature with Alexa Fluor-488 conjugated secondary antibodies (Life Technologies) diluted 1/500 in PBS + 10% FCS. After three washes with PBS + Triton 0.1%, cells were stained with DAPI (final concentration 1 μg/mL) and mounted on microscopy slides with Prolong Gold antifade reagent (Invitrogen). Anti-DHX36 (13159-1-AP) and anti-HSP60 were diluted 1/200 in PBS + 10% FCS. Images were captured with a confocal (Leica DMI6000) microscope. DHX36 enrichment at mitochondria was calculated with the ratio of the DHX36 signal overlapping with HSP60 signal over the total DHX36 signal for each cell on field.

For the lysotracker + mitotracker experiment, live cells were incubated in medium complemented with Lysotracker Deep Red 1/20 000, Mitotracker Green FM 1/10 000 and Hoechst 33342 1/10 000 for 1 h, washed and then observed with a confocal microscope inside a chamber at 37 °C with 5% CO_2_.

### Transmission electron microscopy and immune-gold staining

Samples were fixed by immersion in 2.5% glutaraldehyde and 2.5% paraformaldehyde in cacodylate buffer (0.1 M, pH 7.4), washed in cacodylate buffer for a further 30 min. The samples were postfixed in 1% osmium tetroxide in 0.1 M cacodylate buffer for 1 h at 4 °C and dehydrated through graded alcohol (50, 70, 90, and 100%) and propylene oxide for 30 min each. Samples were oriented and embedded in Epon 812. Semithin sections were cut at 2 µm, and ultrathin sections were cut at 70 nm (Leica Ultracut UCT) and contrasted with uranyl acetate and lead citrate and examined at 70 kV with a Morgagni 268D electron microscope (FEI Electron Optics, Eindhoven, the Netherlands). Images were captured digitally by a Mega View III camera (Soft Imaging System). Immunogold labeling was performed using AURION immunogold reagents essentially as described: (https://aurion.nl/labeling-protocol/post-embedding/protocol-conventional-reagents/) using the DHX36 and COX IV antibodies.

### RNA extraction and RT-qPCR

Total RNA isolation was performed using TRI Reagent (MRC) and isopropanol precipitation, according to the manufacturer’s protocol. Pelleted RNAs were resuspended in water, and DNA was depleted using the TurboDnase Free Kit (ThermoFisher Scientific). RNA was then reverse transcribed with the Superscript IV reverse transcriptase (ThermoFisher Scientific) following the manufacturer’s instructions. qPCR was carried out with SYBR Green I (Roche) and monitored by a LightCycler 480 (Roche). Target gene expression was normalized using TBP, HBMS, and RPL13A as reference genes. For polysome profiling, normalization was performed using mRNAs encoding GAPDH, TBP and HMBS. Primers for RT-qPCR are listed below.

### Protein extraction and Western blotting

Whole cell extracts were prepared by freeze–thaw technique using LSDB 500 buffer [500 mM KCl, 25 mM Tris at pH 7.9, 10% glycerol (v/v), 0.05% NP-40 (v/v), 16 mL DTT, and protease inhibitor cocktail]. Lysates were subjected to SDS-PAGE, and proteins were transferred onto a nitrocellulose membrane. Membranes were incubated with primary antibodies in PBS + 5% BSA + 0.01% Tween-20 o/n at 4 °C. The membrane was then incubated with HRP-conjugated secondary antibody (Jackson ImmunoResearch, 1/2000) for 1 h at room temperature, and visualized using the ECL detection system (GE Healthcare). Antibodies used are listed below.

### Mitochondria fractionation

Mitochondria were isolated with the Mitochondria Isolation Kit (ThermoFisher Scientific) following the manufacturer’s instructions. Briefly, harvested cells were washed and pelleted, resuspended in buffer A, and incubated 2 min on ice. Buffer B was added for 5 min, vortexing every minute, and diluted with buffer C. Nuclei were pelleted 10 min at 700×*g*, and supernatant centrifuged for 15 min at 3000×*g*. Purified mitochondria were washed in buffer C and lysed in CHAPS 2%.

For mitochondrial protein content analysis after digitonin treatment, mitochondria were purified as described above. Then, purified mitochondria were digested on ice for 15 min with increasing concentrations of Digitonin (Invitrogen) in mitochondria isolation buffer (210 mM Mannitol, 70 mM Sucrose, 1 mM EDTA, 10 mM HEPES and protease inhibitors cocktail). Digested mitochondria were pelleted by centrifugation (13,000×*g* for 10 min), and the supernatant was removed. Digested mitochondria were then lysed, and the protein content was analyzed by SDS-PAGE.

For the trypsin treatment, mitochondria were also prepared following the Mitochondria Isolation kit. Mitochondria were subsequently digested for 20 min on ice with 50 µg/mL Trypsin diluted in mitochondria isolation buffer. Digestion was stopped by adding 120 µg/mL of soybean trypsin inhibitor. Pellets were centrifuged for 10 min at 13,000×*g* at 4 °C, and lysed as described above. For cell swelling conditions, mitochondria were digested with trypsin diluted in HEPES-KOH 20 mM at pH 7.5 instead of the mitochondria isolation buffer.

### Identification of RNAs associated with DHX36

Cells were grown in 15 cm petri dishes, harvested by scraping, resuspended in lysis buffer (20 mM Tris-HCl, pH 8, 200 mM NaCl, 2.5 mM MgCl_2_, 0.05% Triton, DEPC water) supplemented with DTT (1 mM), protease/phosphatase inhibitor cocktail (ThermoFisher Scientific) and RNAsin (ThermoFisher Scientific) and kept on ice for 15 min, pipetting every 3 min. Membranes were pelleted 10 min at 10,000×*g* at 4 °C and the supernatant precleared 1 h at 4 °C with protein G magnetic beads (Invitrogen). Lysate was quantified by Bradford protein quantification assay (Bio-Rad) and incubated overnight on a rotating wheel at 4 °C with 5 µg of the indicated antibodies. Then, 50 µL of resuspended Protein G magnetic beads were added for 3 h to isolate RNA–protein complexes and washed five times in lysis buffer. After final wash, RNA was purified by TRI Reagent + isopropanol precipitation and proteins eluted by boiling beads at 95 °C for 15 min in Laemmli buffer.

For RNA sequencing, RNAs were obtained from 501mel cells expressing a control shRNA or an shRNA targeting LENT following the method described above. RNA profiles were determined by using a 2100 Bioanalyser. rRNAs were depleted with the Ribo-Zero Plus rRNA depletion kit (Illumina), and the libraries were prepared with the library prep mRNA ultralow Smarter kit (Takara). Sequencing was performed on a NextSeq 2000 high-throughput sequencer (Illumina). Analyses were performed as described in the next paragraph.

### Bulk RNA sequencing data

Gene expression in 501mel cells transfected with control or LENT-targeting ASO2 was analyzed by RNA-seq. After sequencing, raw reads were pre-processed in order to remove adapter and low-quality sequences (Phred quality score below 20) using cutadapt version 1.10. and reads shorter than 40 bases were discarded. Reads were mapped to rRNA sequences using bowtie version 2.2.8, and were also removed. Reads were mapped onto the hg19 assembly of the Homo sapiens genome using STAR version 2.5.3a. Gene expression quantification was performed from uniquely aligned reads using htseq-count version 0.6.1p1, with annotations from Ensembl version 75 and “union” mode. Only non-ambiguously assigned reads were retained for further analyses. Read counts were normalized across samples with the median-of-ratios method. Comparisons of interest were performed using the Wald test for differential expression and implemented in the Bioconductor package DESeq2 version 1.16.1. Genes with high Cook’s distance were filtered out, and independent filtering based on the mean of normalized counts was performed. *P*-values were adjusted for multiple testing using the Benjamini and Hochberg method.

### Motif enrichment analysis

To identify RIP-seq genes containing G-quadruplex regions, we first retrieved publicly available RIP-seq data for DHX36 reported by Varshney et al. [18] from GEO (accession: GSE154570). We then performed de novo motif analysis using the MEME-ChIP algorithm on DHX36 RIP retained RNAs, identifying the G-quadruplex motif “CCGCCGCY” and generating the associated probability matrix. Lastly, we used the FIMO algorithm to find this motif in the 5’UTR regions of genes identified in the DHX36 RIP-seq analysis.

### RNA in vitro transcription and purification

Double-stranded DNA molecules (gBlocks) containing LENT WT or LENT ΔG sequences were ordered from Integrated DNA Technologies and used as PCR templates to generate RNA by in vitro transcription. DNA constructs were designed with the T7 RNA polymerase promoter sequence. After run-off transcription, RNAs were purified by denaturing polyacrylamide gel electrophoresis (PAGE) and extracted by the “crush and soak method” as described [19]. For EMSA experiments, the purified RNA transcripts were labeled at their 5′ end by addition of a radioactive cap using the Vaccine Capping Enzyme with the ScriptCap m7G capping system from CELLSCRIPT in the presence of [32 P] αGTP (>6000 Ci/mmol). The 5′-radiolabelled transcript was separated from enzyme and free nucleotides by Bio-Spin 6 Columns (Bio-Rad).

### DHX36 protein expression and purification

The inducible expression plasmid (pDHX36 54-989), encompassing the sequence of the human protein (aa 54 to 898) with a His6-SUMO N-terminus tag [20], was generously provided by Rick Russell. Recombinant protein expression was carried out in BL-21 DE3 Rosetta2 pLysS cells (Merck). Cells were grown at 37 °C until reaching an OD-600 nm value of 0.9. Subsequently, the temperature was lowered to 18 °C, and protein expression was induced by adding 0.5 mM IPTG for 16 h. Cells were harvested by centrifugation, and the pellet was resuspended in lysis buffer [50 mM Tris-HCl, pH 8.0, 1 M NaCl, 10% Glycerol, 10 mM 2-Mercaptoethanol, 1 mM CaCl_2_, 10 µg/mL Dnase I (Merck), 1x Halt-Protease (Pierce)] before being lysed by sonication at 4 °C. The lysate was clarified by centrifugation, and nucleic acids were precipitated using 0.1% polyethyleneimine and removed by centrifugation. The supernatant was then passed through a Ni-NTA column (Protino, Macherey-Nagel) equilibrated with buffer A (50 mM Tris-HCl, pH 8.0, 1 M NaCl, 10% Glycerol). After extensive washing with the equilibration buffer, the protein was eluted with buffer A supplemented with 300 mM Imidazole. Factions containing the protein were treated with ULP Protease at a ratio of 1:500 (W/W) and digested/dialyzed overnight at 4 °C in buffer B (50 mM Tris-HCl, pH 8.0, 350 mM NaCl, 10 mM 2-Mercaptoethanol, 10% Glycerol). The DHX36 54-989 protein, liberated from its N-terminal tag, was separated on a NiNTA column equilibrated with buffer B, with the protein predominantly found in the flow-through fractions. Further purification was accomplished via chromatography on a Heparin affinity column (Hitrap HP, Cytiva). The DHX36 54-989 protein was eluted by a linear gradient from 10 to 500 mM NaCl in buffer (50 mM Tris-HCl, pH 8.0, 10% Glycerol, 10 mM 2-Mercaptoethanol), resulting in a symmetrical peak, and isolated at the end of the gradient. The protein fractions were concentrated in the elution buffer to a final concentration of 7 µM (concentration determined using OD at 280 nm, extinction coefficient 108180 M^−1^cm^−1^), snap-frozen in liquid nitrogen, and stored at −80 °C. The identity of the final purified protein was confirmed by mass spectrometry analysis (performed at Strasbourg-Esplanade Proteomics Facility). Dynamic light scattering (DLS) was used to investigate the solubility of the purified protein.

### EMSA

5’ end labeled LENT WT and/or LENT ΔG RNA (10,000 cpm; <3 nM) and a molar excess of oligo(dT) in 8 μL of milli-Q (Millipore) water were heated for 2 min at 90 °C and chilled on ice for 2 min. After the addition of tenfold concentrated refolding buffer (50 mM MES/NaOH, pH 6, 100 mM KOAc, 1 mM Mg(Oac)_2_ and one unit of Rnasin (Promega)), RNA was renatured for 15 min at RT. 10 µL of RNA was finally incubated 30 min in ice with increasing concentrations of DHX36 (0–125 nM) in twofold concentrated binding buffer (final concentration: 50 mM Tris/HCl pH 8, 150 mM NaCl, 10% glycerol, 10 mM β-mercaptoethanol). Electrophoresis was performed in TBM (89 mM Tris base, 89 mM boric acid and 1 mM Mg(Oac)_2_) buffer at 120 V for 5 h at 4 °C. Results were analyzed by phosphorimaging. Quantitative analysis was performed using ImageLab software (Bio-Rad).

### Sucrose density gradient ribosome profiling

Cells were washed twice with PBS, harvested by scraping and rapidly centrifuged at 300×*g* for 5 min at 10 °C. The resulting pellet was resuspended in lysis buffer (20 mM HEPES/NaOH, pH 7.4, 100 mM KOAc, 1 mM DTT, 0.5 mM Mg(Oac)2, 100 U of Recombinant Rnasin (Promega) and Halt Protease inhibitor cocktail (ThermoFisher)). Cell lysis was performed by nitrogen cavitation with 4639 Cell Disruption Vessel (Parr Instrument Company) at 350 psi for 50 min, stirring with a small magnet at 500 rpm in a cold room (4 °C). Lysate was then centrifuged at 1000×*g* for 5 min, and the supernatant was recovered, avoiding the foam (membranes) and the pellet (nuclei). After an incubation of 5 min at 30 °C, 20–30 OD260 of cell extracts from both cell lines were gently layered over 7–47% sucrose gradients in buffer T (25 mM Hepes/NaOH, pH 7.4, 79 mM KOAc, 2.5 mM Mg(Oac)_2_, 1 mM DTT, 3 U/µL Rnasin (Promega)). Gradients were centrifuged at 37,000 rpm (Beckman, SW41Ti) for 2 h and 30 min at 4 °C. After centrifugation, 45 fractions (0.25 mL/fraction) were collected on a BIOCOMP gradient fractionator equipped with a UV detector. For RNA-seq, biological triplicate ribosome profiling was performed from control or shLENT-silenced cells as described above. RNA from three fractions corresponding to the 80S, light or heavy polysome fractions was pooled and sequenced using the Ribo-Zero Plus rRNA depletion kit (Illumina) as described above.

### Statistics

All tests used for statistical significance were calculated using GraphPad Prism 10 and indicated in the figure legends along with *P*-values (****P < 0.0001, ***P < 0.001, **P < 0.01, *P < 0.05, ns: *P* > 0.05). At least 3 biological replicates were performed for cell-based experiments and 6 for CDX tumor experiments. No samples were excluded from cell-based assays. No animals were excluded from the CDX cohorts. Data showed expected variation, and variance was comparable between the compared groups. All error bars represent ±SD.

### Resources

All oligonucleotides and antibodies used are listed in Tables 1–4 below.

**Table 1:** Antisense (ASO) and short hairpin oligonucleotides (shRNA) targeting the indicated transcripts.

GapmeR/siRNA/shRNA	Sequence
ASO NEG	AACACGTCTATACGC
ASO LENT 1	TTTGATGAGTGAGTCG
ASO LENT 2	GAGTCGCTGAGAATTA
ASO LENOX	GTAGAGGCTAGAACTG
ASO SAMMSON	GTGTGAACTTGGCT
shSCR	ATTACGTCTGTCATGAACCTC
shLENT	CCTTCCAAGCATTGCCTTTAT
siCTRL	UGGUUUACAUGUUGUGUGA
siDHX36	CGGCAUGUGGUACGCGAAA

**Table 2:** Primers—oligonucleotides used for RT-qPCR.

RT-qPCR primers	Sequence
GAPDH_F	ACAACTTTGGTATCGTGGAAGG
GAPDH_R	GCCATCACGCCACAGTTTC
RPL13a_F	TTGAGGACCTCTGTGTATTTGTCAA
RPL13a_R	CCTGGAGGAGAAGAGGAAAGAGA
HMBS_F	GGCAATGCGGCTGCAA
HMBS_R	GGGTACCCACGCGAATCAC
TBP_F	CGGCTGTTTAACTTCGCTTC
TBP_R	CACACGCCAAGAAACAGTGA
LENOX _F	ACCTAACCTGCGAATGCTGT
LENOX _R	GCCTAAACATTTGCTGCCCC
LENT_F	CAATGCTTGGAAGGCGTGAT
LENT_R	AAACGTATGGCCACCTCTGA
MALAT1_F	GGATTCCAGGAAGGAGCGAG
MALAT1_R	ATTGCCGACCTCACGGATTT
UBE4A_F	GAGAGCCAAGGAAGAGATTACCA
UBE4A_R	CTTGTTCATGTACTCACGGGC
RBPJ_F	GGAAAGAGCAAAGGAGGGGA
RBPJ_R	TCACCAAATTTCCCAGGCGA
NOX4_F	CACCAGATGTTGGGGCTAGG
NOX4_R	CTCCTGGTTCTCCTGCTTGG
SDHB_F	AGGATCTTGTTCCCGATTTGAG
SDHB_R	CGTAGAGCCCGTCCAGTTTC
ATP5A_F	CTGCAAAGATGCTGTCCGTG
ATP5A_R	GCATTTCTGGAGACCAGTCC
UQCRC2_F	CCAAGCTGCCAAGAACAAGC
UQCRC2_R	CAGCAACTAGAGCCTGGGAC

*F* forward strand, *R* reverse strand.

**Table 3:** Antibodies used in this study indicating host type, application (WB, IF, IP), dilution and lot number.

Primary antibodies	Host	Application	Dilution	Lot number
DHX36 (13159-1-AP)	Rabbit	WB IF IP	1/1000 1/200 5 µg/mL	00004147
CARF (BE-A303-861A-M)	Rabbit	WB	1/1000	1
RAP2 (sc-515711)	Mouse	WB	1/1000	B4397
HSP60 (in-house)	Mouse	WB IF	1/500 1/100	4MTE-2H7
VINCULIN (V4505)	Mouse	WB	1/5000	099M4850V
ACTIN (in-house)	Mouse	WB	1/1000	1ACT-2D7
COX IV (ab202554)	Rabbit	WB	1/1000	GR3342068-1
UBE4A (sc-365904)	Mouse	WB IF	1/1000 1/200	C3117
NOX4 (NB110-58849)	Rabbit	WB IF	1/1000 1/200	D134519
EIF4A2 (ab31218)	Rabbit	WB	1/1000	GR3383097-1
RPL36 (PA5-117106)	Rabbit	WB	1/2000	WG3327290E
C1QBP (A302-863A) = p32	Rabbit	WB IF	1/1000 1/200	1
TOMM20 (H00009804-M01)	Mouse	WB	1/1000	HC5202153D
RBP-Jk (sc-271128)	Mouse	WB IF	1/500 1/100	A2220
OXPHOS (45-8199)	Mouse	WB	1/1000	VB2939036
LC3B (ab51520)	Rabbit	WB	1/5000	GR3374012-2
gH2AX (ab22551)	Mouse	WB IF	1/1000 1/100	GR3358071-2
FUNDC1 (NBP1-81063)	Rabbit	WB	1/500	000042870
CTSD (2284)	Rabbit	WB	1/1000	2
WFS1 (26995-1-AP)	Rabbit	WB	1/500	00096359
HSPA5 (HPA038845)	Rabbit	WB	1/500	A83196
Normal Rabbit IgG (12-370)	Rabbit	IP	5 µg/mL	3493998

*WB* western blot, *IF* immunofluorescence, *IP* immunoprecipitation.

**Table 4:** Sequences of biotinylated oligonucleotides used for RNA pulldown experiments.

RNA pulldown probes	Sequence
PCA3-1	GCACTTGCTATTTCTTCTGT
PCA3-2	CTCTGTTTTTCTGATGCCAG
PCA3-3	TGTTTGTTGCATGTCTTGTG
PCA3-4	ATTCTTTATTGCCAGGAGTG
PCA3-5	TATGCATATTGTGGTTGTCC
PCA3-6	TGTCTGAATCCTCTCCAAAC
PCA3-7	GCTAGCATCCATAATAGGAG
PCA3-8	TTGCATGCATGTACCACAAG
LENT-1	GAAATGTACACCATGCTGGG
LENT-2	TTATTTTGCTCCTTGCTGTT
LENT-3	TGAGACCCCAAAGAGGGAAA
LENT-4	CCTTGCTGTTCTCGAAAGAT
LENT-5	CCTGGCTTTGATGATTCAGT
LENT-6	GGCAATGCTTGGAAGGCG
LENT-7	TTGCCACCAATCTCTCTG
LENT-8	GCGTGATAAGCTACCCAG
LENT-9	CAGAGGTGGCCATACGTTTG
LENT-10	GCTTGATGGGGAGAAGGAAG

## Results

### LENT is expressed in melanocytic melanoma cells and is associated with poor patient outcome

Integration of MITF and SOX10 ChIP-seq data with RNA-seq following MITF silencing in 501Mel melanocytic melanoma cells [9] identified LENT (LINC00520) as an lncRNA directly and positively regulated by MITF. LENT expression was reduced upon silencing of MITF or its cofactor BRG1, and the corresponding locus displayed several MITF and BRG1-bound sites and was marked by H3K27ac in 501Mel cells (Fig. S1A). LENT expression was low in normal tissues (0.854 Log2 normalized counts) with the highest expression in the esophagus mucosa and stomach in the GTEX database (Fig. S1B and data not shown). Expression was highest in cutaneous melanoma (SKCM, 6.189 Log2 normalized counts), compared to other cancer types (1.490), including uveal melanoma (UVM, 1.732) (Fig. S1B). Expression was also upregulated in primary melanoma compared to benign nevi and normal tissues (Fig. S1C). Thus, LENT expression was negligible in normal tissues and upregulated more than 60-fold in cutaneous melanoma.

Analyses of scRNA-seq data from human melanoma xenografts [3] showed that LENT was widely expressed except in NCSC and mesenchymal cells (Fig. S1D), while in scRNA-seq data from human melanoma patients [7], it was also broadly expressed except in mesenchymal cells and was strongest in the hypoxia-stress cell cluster (Fig. S1E). Preferential LENT expression in melanocytic type cells was confirmed by RT-qPCR analyses of a collection of melanoma cell lines (Fig. S1F). Cytoplasmic LENT localization in MITF-expressing melanoma cells could be directly observed using RNA-scope on human melanoma patient sections, whereas its expression was low in normal melanocytes (Fig. S1G). RNA-scope also showed a predominantly cytoplasmic localization in cultured melanoma cells, whereas no signal was seen in HeLa cells (Fig. S1H). Further analyses showed the 432 bp isoform 5 as the most abundant, but not exclusively expressed isoform, in 501Mel cells and also in melanoma patients (Fig. S2A–C). LENT is therefore a cytoplasmic melanoma-enriched lncRNA most abundant in melanocytic MITF-expressing cells.

To determine whether LENT expression correlated with patient outcome, we divided the TCGA-SKCM dataset into primary and metastatic samples, performed unsupervised clustering of the transcriptome data from each and GSEA analyses of differentially expressed genes to define the signatures of each cluster. In primary melanoma, LENT was co-expressed with MITF and SOX10 in cells defined by an oxidative phosphorylation (OxPhos) and cell cycle signature typical of melanocytic MITF-expressing cells, but was strongly reduced in mesenchymal (designated as EMT, epithelial to mesenchymal transition) cells expressing markers such as PRRX1 (Fig. S3A). In contrast, as previously described [15], LENOX displayed a broader expression pattern, also being expressed in EMT cells. In metastatic melanoma, LENT again was strongest expressed in the MITF-SOX10 expressing OxPhos/cell cycle cells, but reduced in cells with EMT signatures (Fig. S3B).

In primary melanoma, high LENT expression was associated with better survival, whereas in metastatic samples, high LENT expression was associated with poorer outcome (Fig. S3C, D). These observations are in agreement with and extend previously published analyses [21] and are in line with the idea that low LENT-expressing mesenchymal cells promote metastases of primary melanoma [6], whereas LENT-expressing cells with OxPhos and cell cycle signatures associate with poorer survival in metastatic samples, hence accounting for the differential association of LENT expression with survival.

### LENT cooperates with LENOX and SAMMSON to promote melanoma cell proliferation and survival

**Fig. 1:**
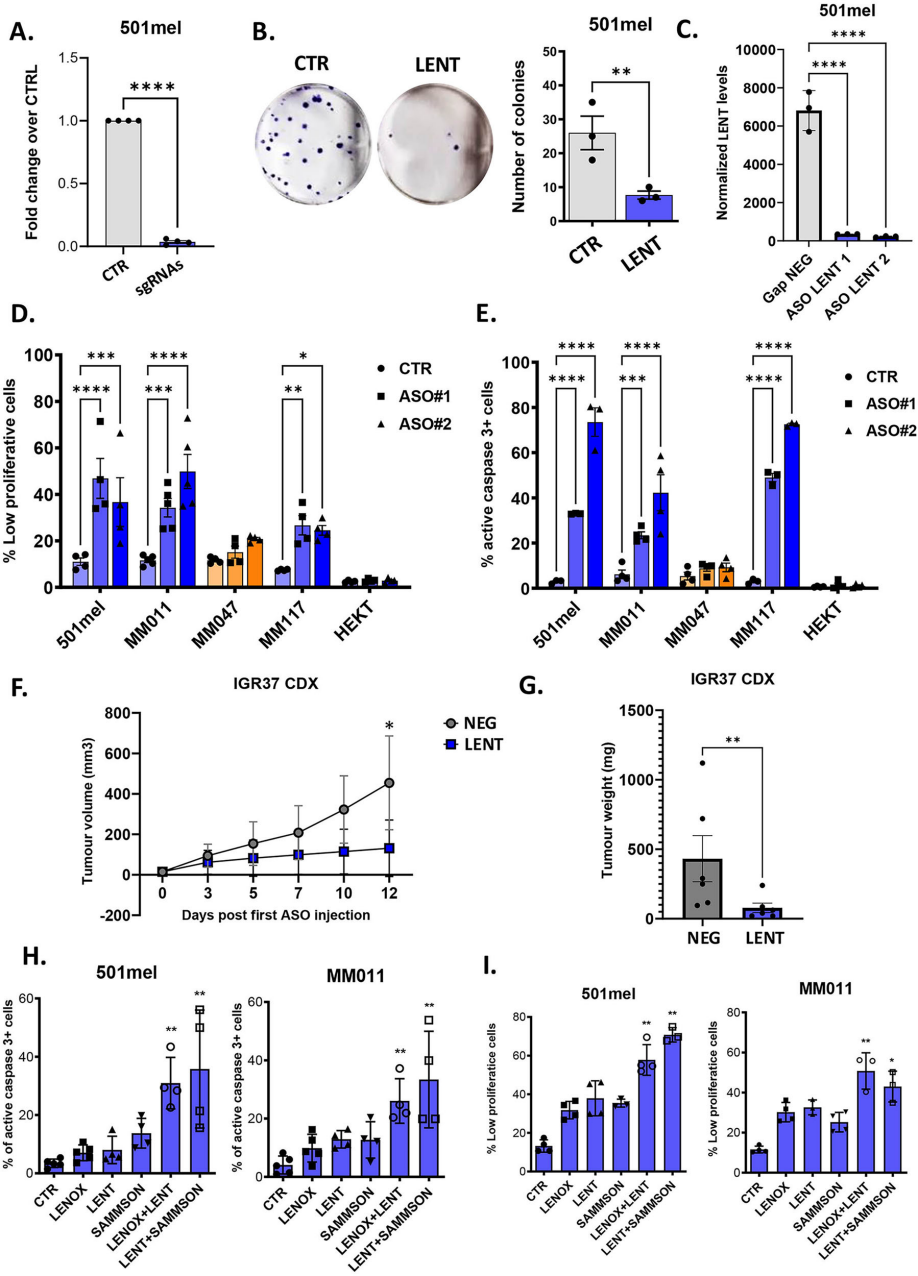
LENT depletion impairs melanoma cell proliferation and survival. **A** LENT levels measured by RT-qPCR after dCas9-KAP1-mediated LENT silencing compared by paired *t*-test. **B** Colony formation assay upon dCas9-KAP1-mediated LENT silencing, compared by one-way ANOVA (Dunnett test). **C** LENT levels measured by RT-qPCR after ASO-mediated depletion with two independent ASOs. **D**, **E** Measurement of slow proliferating or apoptotic cells by flow cytometry upon ASO-mediated LENT depletion compared by one-way ANOVA (Dunnett test). Melanocytic cell lines are represented in blue, and mesenchymal cells are colored in orange. **F**, **G** Tumor volumes in mice with IGR37 CDX tumors were measured at the indicated number of days following initial injections of LENT-targeting ASO. Tumors were weighed following sacrifice at day 14. Following the first ASO injection. Volumes were compared by two-way ANOVA and weights by Mann–Whitney test. **H**, **I** Same measurements as for D-E but after ASO-mediated depletion of the indicated lncRNAs at sub-optimal ASO doses, compared by one-way ANOVA (Dunnett test). *P < 0.033; **P < 0.0021; ***P < 0.0002; ****P < 0.0001.

To address the function of LENT in melanoma cells, we silenced its expression by CRISPR interference (CRISPRi) using dCAS9-KAP1, transfection of locked nucleic acid GapmeR antisense oligonucleotides (ASO) or by Doxycycline (Dox)-inducible expression of LENT-targeting shRNA. CRISPRi silencing in 501Mel cells with LENT promoter-targeting sgRNAs that potently reduced its expression resulted in strongly reduced colony-forming capacity (Fig. 1A, B). Transfection of melanoma cells with different phenotypes and driver mutations, with 2 independent ASOs that reduced LENT expression by over 80% compared to a non-targeting control (CTR) (Figs. 1C, S2D, E) and reduced the growth of melanocytic, but not mesenchymal melanoma cells nor HEK293T cells that did not express LENT (Fig. S4A). ASO-mediated silencing resulted in strongly reduced cell proliferation (Fig. 1D) and a strong increase in cleaved caspase 3-expressing apoptotic cells (Fig. 1E), with early and late apoptotic cells observed in flow cytometry (Fig. S4B). We also silenced LENT with a stably integrated Dox-inducible shRNA that efficiently reduced 501Mel proliferation (Figs. S2E, S4C). In contrast, ectopic Dox-induced expression of LENT isoform 5 stimulated colony formation in melanoma cells, but also in HEK293T cells, where it was not normally expressed (Fig. S4D, E).

To test if LENT silencing could also block xenograft tumor growth, melanocytic IGR37 cells were injected subcutaneously in immunodeficient mice, and when tumors reached ≈100 mm^3^, mice were subsequently injected subcutaneously every 2 days with LENT ASO. Compared to untreated controls, injection of LENT ASO reduced LENT expression in tumors and strongly reduced tumor growth and tumor weight, but did not affect animal body weight and therefore did not show toxicity at least over the time period of the experiment (Figs. 1F, G, S4F).

All 3 targeting strategies, as well as gain of function, therefore revealed the essential role of LENT in the proliferation and survival of melanocytic melanoma cells in culture and in vivo xenografts, similar to previous reports [21].

We previously showed that ASO silencing of LENOX and SAMMSON cooperated to induce melanoma cell death [15]. To assess if LENT also collaborated with LENOX and SAMMSON, melanocytic 501Mel and MM011 cells were transfected with sub-optimal concentrations of ASO targeting LENT alone or together with LENOX or SAMMSON. Each ASO specifically targeted its cognate lncRNA target without affecting expression of the others, with the exception of SAMMSON, which was upregulated in the LENT-LENOX targeted MM011 cells (Fig. S5A). Compared to LENT, LENOX or SAMMSON alone, a cooperative increase in apoptosis of both lines was observed using the combinations of ASO, and an additive increase in slow proliferation (Fig. 1H, I). LENT silencing also cooperated with MAP Kinase inhibition by the dabrafenib and trametinib combination to eradicate melanoma cells (Fig. S5B). MITF and SOX10, therefore, coordinately regulate a network of 3 lncRNAs that cooperate to promote melanoma cell survival.

### LENT interacts with the G4 resolvase DHX36

To investigate the molecular pathways regulated by LENT, we performed RNA-seq from ASO-control or LENT ASO2-silenced cells. This experiment revealed 97 upregulated and 82 down-regulated transcripts (Log2 fold-change ± 1, *P* < 0.05) (Fig. S6 and Dataset S1). However, as LENT is predominantly cytoplasmic, we reasoned that a direct effect of LENT on transcription would be unlikely.

**Fig. 2:**
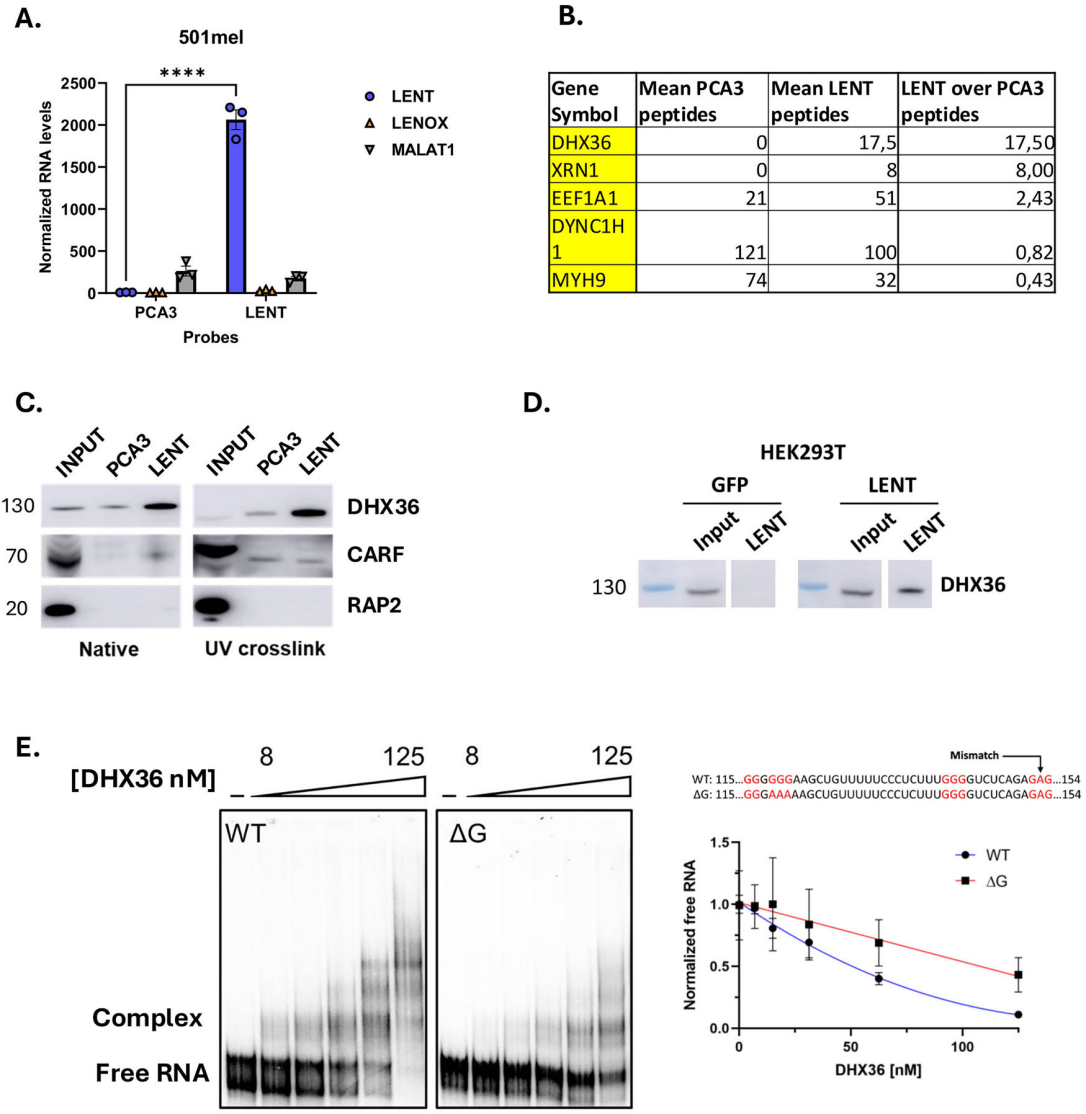
LENT interacts specifically and directly with the G4 resolvase DHX36. **A** Measurement of lncRNA levels by RT-qPCR after RNA pulldown with control probes (PCA3) or LENT-targeting probes by two-way ANOVA. **B** LC/LC Mass spectrometry analysis of peptides retrieved after biological triplicate pulldown of PCA3 or LENT. **C** Immunoblot showing protein enrichment after the indicated RNA pulldowns, in native or UV-crosslinked conditions. **D** Native LENT pulldown upon ectopic expression of GFP or LENT in HEK293T cells. **E** EMSA assay performed with T7 in vitro transcribed WT or mutated LENT in the presence of increasing concentrations of purified truncated DHX36. The potential G4-forming structure predicted by PQSfinder in LENT isoform 5 sequence is indicated in red. The mutated LENT sequence used for the EMSA with the three guanines mutated in adenines underlined. *P < 0.033; **P < 0.0021; ***P < 0.0002; ****P < 0.0001.

To investigate other potential mechanisms, we sought to identify LENT-interacting proteins. We performed pulldown from cytoplasmic extracts of 501Mel cells using a tiling array of biotinylated oligonucleotides complementary to LENT or as a negative control, the prostate cancer lincRNA PCA3, followed by mass spectrometry. Compared to LENOX or MALAT1, LENT was selectively enriched using its cognate oligonucleotides, but not those of the PCA3 control (Fig. 2A). Triplicate purifications were performed, and LENT-interacting proteins were identified by mass spectrometry. DHX36 was the most enriched protein in the LENT pulldown, with no peptides found in the 3 control samples, but an average of 17 in the LENT pulldowns (Fig. 2B and Dataset S2). To confirm this interaction, we performed LENT pulldown from native or UV-crosslinked extracts followed by immunoblot. Under both conditions, DHX36 was enriched in the LENT pulldown compared to the PCA3 control, whereas neither the SAMMSON-interacting CARF [14] nor LENOX-interacting RAP2 [15] was enriched (Fig. 2C). For further confirmation, we performed LENT pulldown from the HEK293T cells ectopically expressing LENT isoform 5. DHX36 was detected after pulldown from LENT-expressing HEK293T cells, but not from control cells with an empty GFP vector (Fig. 2D).

To ask if LENT interacts directly with DHX36, we generated and purified recombinant DHX36 in *E.Coli* (Fig. S7A, B) and performed electrophoretic mobility shift assay (EMSA) with in vitro transcribed LENT isoform 5 RNA (Fig. 2E). The presence of increasing amounts of purified DHX36 shifted LENT into slower migrating DHX36-RNA complexes. As DHX36 binds RNA with G4 structures [22], we used the QGRS program [23] that predicted a potential G4 structure in LENT, but with a rather low score. Mutation of three guanines in this sequence decreased complex formation in EMSA, but did not fully abolish the interaction (Fig. 2E). Together, these in cellulo and in vitro experiments revealed a selective and direct interaction of LENT isoform 5 with DHX36 that is partially dependent on a potential G4-forming sequence in LENT.

### LENT modulates the association of mRNAs with DHX36

**Fig. 3:**
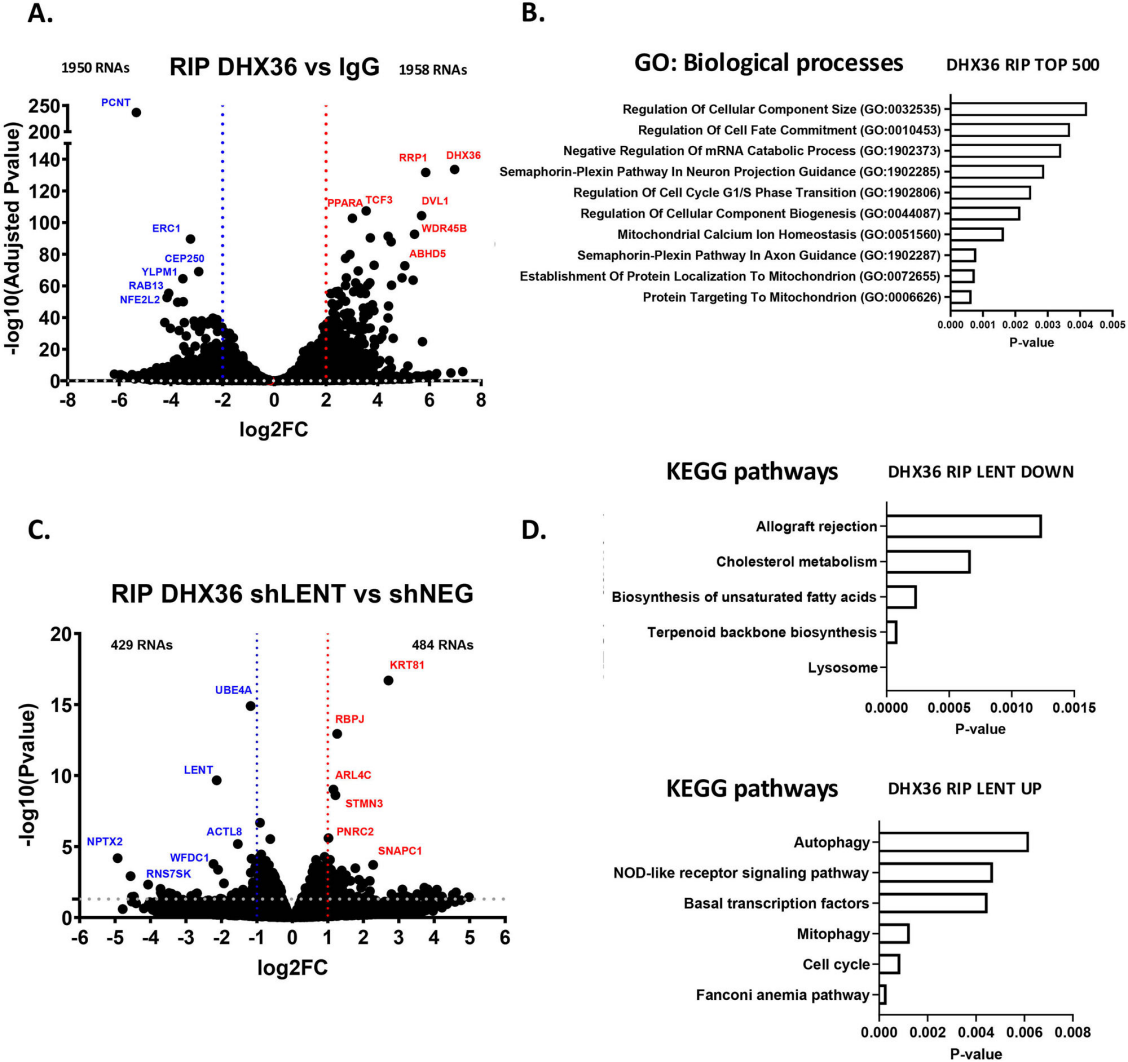
LENT modulates the association of RNA with DHX36. **A** Volcano blot showing RNAs enriched or depleted in the DHX36 IP vs control IgG IP. *P*-values were derived using the Wald test and adjusted using Benjamini–Hochberg FDR correction. **B** Gene ontology analysis by EnrichR software of the 500 most enriched RNAs in the DHX36 IP. **C** Volcano blot showing RNAs enriched or depleted in the DHX36 IP upon shRNA-mediated LENT silencing. *P*-values were derived using the Wald test. **D** KEGG pathways by EnrichR of RNAs enriched or depleted in DHX36 IP upon LENT depletion.

DHX36 unwinds G4 structures in both DNA and RNA, and in particular in the 5’-UTR of mRNAs to facilitate their translation [22, 24–27]. This observation suggested that LENT may modify DHX36 interactions with mRNAs and their translation in melanoma cells. We therefore investigated the mRNAs associated with DHX36 and determined if their association was modulated by LENT silencing. We performed triplicate DHX36 or control IgG immunoprecipitations (IP) from 501Mel cells expressing control shRNA, and the associated mRNAs were sequenced to identify those associated with DHX36 in control conditions. Almost 2000 transcripts were enriched in the DHX36 IP compared to IgG, whereas 1949 were less present in the DHX36 IP compared to control (Log2 fold-change ± 1, *P* < 0.05) (Fig. 3A and Dataset S3). One of the most enriched was the DHX36 mRNA, suggesting DHX36 acts to regulate its own translation in a positive regulatory loop. Ontology analysis showed that DHX36-associated mRNAs were enriched in those encoding proteins involved in mitochondrial function with protein targeting to mitochondrion, mitochondrial calcium ion homeostasis among the most enriched terms (Fig. 3B). Comparison with RNA-seq data from 501Mel cells showed no correlation between association with DHX36 and expression levels excluding the possibility that we spuriously enriched highly expressed mRNAs in the DXH36 IP (Fig. S8A).

It has been reported that RNAs that associate with DHX36 are enriched in a GG-rich motif with a propensity to form G4 structures ([28] and Fig. S8B). This motif was predicted to be present in around 20% of the RNAs enriched in the control IP, but close to 40% in the DHX36 IP (Fig. S8C) and increased to 50–60% when considering the mRNAs most enriched in the DHX36 IP (Fig. S8C and Dataset S3). Examination of the DHX36 mRNA sequence with QGRS mapper indeed identified several potential G4-forming sequences, including the 5’-GGnGGnGG-3’ motif (Fig. S8D), consistent with the observation that it was one of the most enriched mRNAs. We compared RNAs enriched in the DHX36 IP in 501Mel cells with previously published RNA-seq data from HeLa or HEK293T cells designed to identify G4-containing RNAs [29, 30]. Comparing the overlap between the 2 HeLa datasets showed 1832 common RNAs representing between 46% and 64% of the identified G4-containing RNAs using different techniques. Comparison with the 501Mel RNAs enriched by DHX36 IP showed that 900 (46%) were shared with the HeLa and HEK293T datasets, with 341 common to all (Fig. S8E). These common RNAs were enriched in terms associated with transcription and MAP Kinase signaling (Fig. S8F).

To investigate if LENT silencing modified mRNA interaction with DHX36, we directly compared RNA-seq of the DHX36 IP from the control shRNA with the DHX36 IP from the shLENT cells and identified 484 transcripts displaying increased association with DHX36 in the absence of LENT and 429 with less association (Fig. 3C, and Dataset S3). As expected, due to its downregulation by shRNA silencing, LENT was identified as less associated with DHX36. Ontology analyses of mRNAs showing increased DHX36 association revealed enrichment in several process including cell cycle, mitophagy and autophagy, whereas those less associated were enriched in lysosome, metabolic process and allograft rejection (Fig. 3D). Analyses of the RNAs whose association with DHX36 was affected with the QUADRatlas software showed that a large majority comprised experimentally described and/or predicted G4s (Fig. S8G). Together, these data define DHX36-associated RNAs in melanoma cells and identify RNAs whose association with DHX36 was positively or negatively modulated by LENT.

### LENT and DHX36 are associated with the ribosome and regulate coordinated engagement of mRNAs encoding proteins involved in ER homeostasis with polysomes

**Fig. 4:**
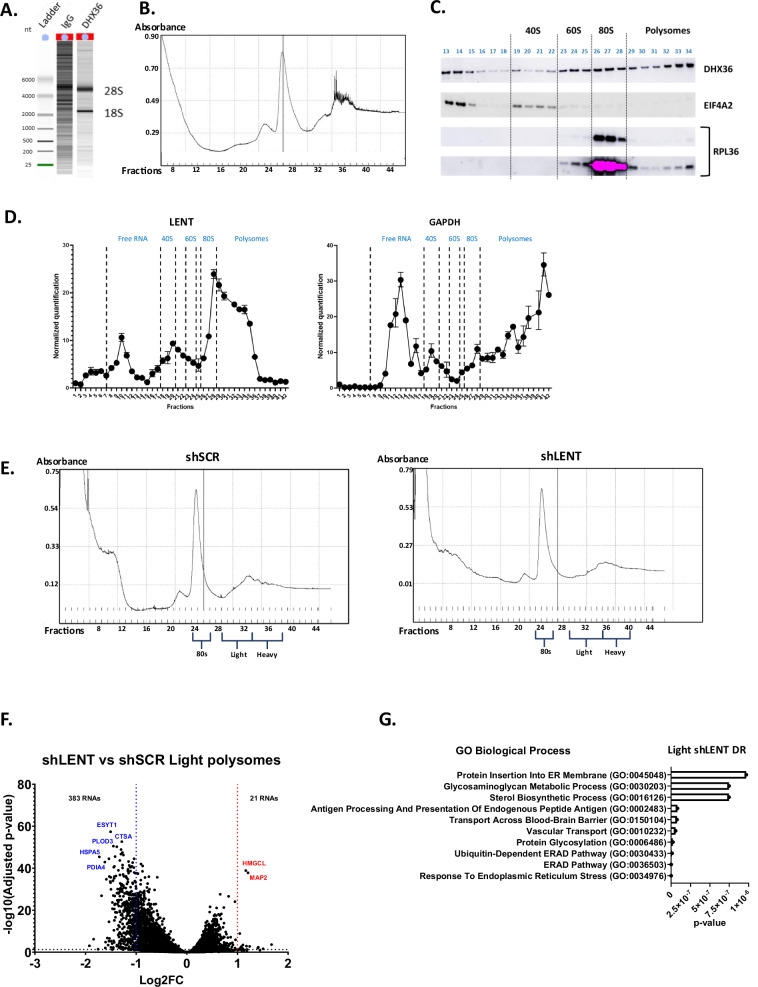
LENT regulates polysome engagement of a subset of mRNAs. **A** Bio-analyzer analyses of precipitated RNA show strong enrichment of 18S and 28S rRNAs in the DHX36 IP but not the control IgG IP. **B** Sucrose gradient separation of ribosomes with rRNAs measured by UV-light spectrometry. **C** Immunoblots for DHX36, the initiation factor EIF4A2 and the large ribosomal subunit RPL36 after polysome separation. EIF4A2 is enriched in the 40S and RPL36 in the 80S and heavier polysome fractions. **D** Presence of LENT or GAPDH mRNA in fractionated ribosomes as measured by RT-qPCR. **E** Representative sucrose gradient separation of ribosomes with rRNAs measured by UV-light spectrometry. Fractions pooled for the 80S, LP and HP are indicated. **F** Volcano blot showing RNAs enriched or depleted in the LP fraction from control or shLENT cells. **G** Gene ontology analysis by EnrichR software of the 383 depleted RNAs in the shLENT LP fraction.

While preparing the DHX36-associated RNAs for sequencing, we noted that the 28S and 18S rRNAs were strongly enriched in the DHX36 IP, but not the control IP (Fig. 4A), and therefore used ribo-depletion kits to prepare the libraries for RNA-seq. This observation, however, strongly suggested that DHX36 was associated with the ribosome. To assess this, we performed polysome profiling of 501Mel cell extracts and analyzed both RNA and protein contents of the fractions. Based on the RNA absorption profile (Fig. 4B) and the distribution profiles of EIF4A2 (initiation factor marking the 40S) and RPL36 (component of the large subunit), we designated the 40S, 60S, 80S and polysome fractions. DHX36 showed association with the 60S, 80S and was additionally present in the light polysome fractions (Fig. 4C). As expected, the control GAPDH mRNA was enriched in the heavier polysome fractions, whereas LENT showed a strong peak in the 80S fraction, but rapidly decreased in the light polysome fractions (Fig. 4D). These observations suggested that LENT may associate with DHX36 on the 80S and light polysome fractions.

To investigate if the LENT-DHX36 axis regulated mRNA association with ribosomes, we prepared biological triplicate polysome fractions from shControl or shLENT-silenced 501Mel cells. We pooled RNA from four fractions representing the 80S, light or heavy polysome components from each replicate and assessed their composition by RNA-seq (Fig. 4E and Dataset S4). Few RNAs showed differential presence in the heavy polysome (HP) fractions from the control or LENT-silenced cells, whereas 184 and 246 transcripts were depleted or enriched, respectively, in the 80S fraction (Log2 fold-change ± 1, *P* < 0.05) (Fig. S9A, B and Dataset S4). However, the most striking effect was seen in the light polysome (LP) fractions, where 383 transcripts were depleted in the LENT-silenced cells, while only 21 were enriched (Fig. 4F).

**Fig. 5:**
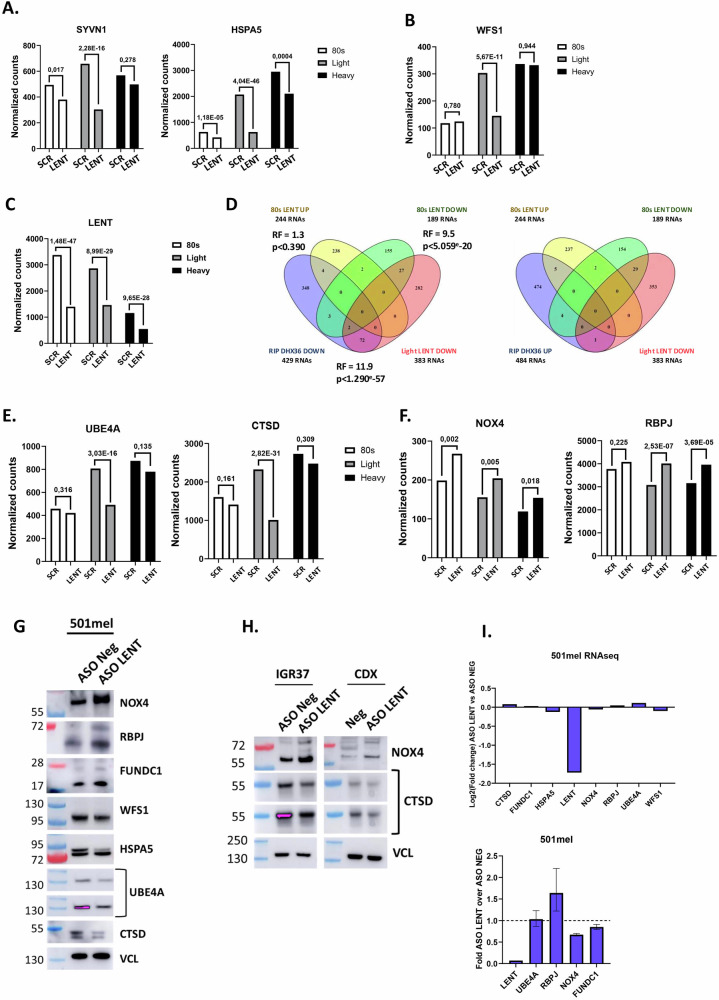
LENT fine-tunes translation of mRNAs differentially associated with polysomes. **A**–**C** RNA-seq data showing the representation of LENT in the 80S, LP and HP fractions. The normalized number of reads is shown along with the adjusted *P*-value between the control and shLENT conditions. **D** Venn diagrams comparing RNAs modulated in the DHX36 RIP in the presence or absence of LENT silencing with those differentially present in the 80S and LP fractions. Significant Representation Factors are shown. **E**, **F** RNA-seq data showing the representation of the indicated RNAs in the 80S, LP and HP fractions. The normalized number of reads is shown along with the adjusted *P*-value between the control and shLENT conditions. **G** Immunoblots of the indicated proteins 48 h following ASO-mediated LENT silencing in 501Mel cells. **H** Immunoblots of the indicated proteins 48 h following ASO-mediated LENT silencing in the IGR37 cell line or extracts from IGR37 CDX tumors from control or LENT ASO-injected mice. **I** Expression of the indicated RNAs in ASO control or ASO LENT-transfected cells represented as fold-change in RNA-seq (upper panel) or RT-qPCR (lower panel).

Ontology analyses of the 383 RNAs depleted in the LP fractions revealed their strong enrichment in several pathways pertaining to endoplasmic reticulum (ER) homeostasis, such as ER stress, ER-associated protein degradation (ERAD), protein glycosylation and cholesterol metabolism (Fig. 4G and Dataset S5). KEGG ontology analyses gave comparable results, but further revealed enrichment in lysosome function (Dataset S5). RNAs encoding key components of the ER stress/ERAD pathways, such as the E3 ligase SYNV1, the HSPA5 chaperone, and the PDIA3, -4, and -6 enzymes, were all significantly depleted in the LP fractions, with PDIA encoding mRNAs also depleted in the 80S fraction (Figs. 5A, S9C). The mRNA encoding WFS1 involved in ER Ca^2+^ transport was also depleted in the LP fractions, along with those encoding the NOMO1, -2 and -3 proteins and other components of the multi-pass translocon complex (Fig. 5B and Dataset S5). Similarly, mRNAs encoding the DPAGT1 and GALNT2, 7 and 12 enzymes involved in N-linked or O-linked protein glycosylation, respectively, were all depleted in the LP fractions (Dataset S5). Related to the above, mRNAs encoding the MHC class 1 HLA-A, -B and -C antigens, as well as the TAP1, TAP2 and CALR proteins involved in their transport and antigen presentation, were depleted in the LP fractions (Fig. S9D, E and Datasets S4, S5). Overall, these transcripts showed no bias toward low expression compared to the overall transcriptome, but rather were well expressed. Furthermore, they displayed no bias in length compared to the overall transcripts of the LP fraction, although, as may be expected, transcripts in the LP were significantly longer than those in the 80S fraction (Fig. S10A, B). These results indicated that engagement in LPs of mRNAs encoding key components of many processes associated with normal ER homeostasis and/or intracellular protein transport was coordinately regulated by LENT.

We interrogated the polysome RNA-seq to determine if the mRNAs whose association with DHX36 was positively or negatively regulated by LENT were also differentially engaged with the polysome fractions. Depletion of LENT was clearly seen in all fractions (Fig. 5C). Of the 383 RNAs depleted in the LP fractions, 74 were also depleted in the DHX36 RIP upon LENT silencing (Fig. 5D and Dataset S4), representing a highly significant, but incomplete overlap between the 2 experimental approaches. In contrast, almost no RNAs showed discordant regulation, with only a single transcript up in DHX36 RIP and down in the LP fractions. Moreover, a smaller, but significant, overlap (29 transcripts) was seen with the transcripts depleted in the 80S fraction, with only 4 discordant transcripts. Ontology analyses of the 74 common mRNAs revealed their strong enrichment in ER homeostasis, including the above-mentioned HLA proteins, and lysosome function analogous to the 383 LP-depleted transcripts (Fig. S10C and Datasets S4, S5). Similarly, analyses of the RNAs depleted in the 80S fraction using relaxed criteria of Log2 fold-change ≥0.7, but with a more stringent adjusted *P*-value of <0.01, also showed a strong enrichment in many of the same terms related to ER homeostasis (Dataset S5).

Together, these analyses showed that the effect of LENT depletion on mRNA association with both the 80S and LP fractions was neither random nor general, but selectively affected transcripts with specific functions that displayed a highly significant, but incomplete overlap with those whose association with DHX36 was promoted by LENT. We note that these transcripts do not overlap with those whose expression was altered upon LENT silencing (Fig. S6).

QUADRatlas analyses of the 383 LP-depleted mRNAs indicated the presence of experimentally defined (342/368) and predicted (217/368) G4-forming sequences (Fig. S10D). This was consistent with the idea that the LENT-DHX36 axis regulated their unwinding to facilitate their translation. To test this, we investigated whether mRNAs whose association with DHX36 and/or engagement with the LP fractions was modified by LENT silencing were differentially translated. The mRNAs encoding UBE4A and CTSD, whose interaction with DHX36 was reduced upon LENT silencing, also showed reduced association with 80S, LP and HP fractions, with the strongest reduction in the LP fraction (Fig. 5E). UBE4A and CTSD accumulated to lower levels following ASO-mediated LENT silencing, whereas RNA-seq and/or RT-qPCR showed no change in overall abundance of the corresponding mRNAs (Fig. 5G, I). The decreased protein level was therefore most likely due to altered translation.

In contrast, mRNAs encoding the ROS generating enzyme NOX4 known to promote melanoma [26, 31] and RBPJ whose association with DHX36 was increased upon LENT silencing were also increased the 80S, LP and HP fractions and although the fold change was below cutoff, their increased association was statistically significant (Fig. 5F). Protein levels of NOX4, FUNDC1 and RBPJ were increased upon ASO-mediated LENT silencing with again no overall changes in the corresponding mRNA levels (Fig. 5G, I). Furthermore, increased NOX4 and reduced CTSD levels were seen in extracts from IGR37 xenograft tumors treated with LENT ASO (Fig. 5H). Similarly, levels of HSPA5 and WFS1 proteins whose RNAs were less associated with the LP fractions were also decreased despite the fact that we did not detect changes in their association with DHX36 (Fig. 5G). These data showed that LENT modulated interactions of mRNAs with DHX36 and/or the ribosome LP fractions to regulate their translation.

### LENT and DHX36 are enriched in mitochondria

**Fig. 6:**
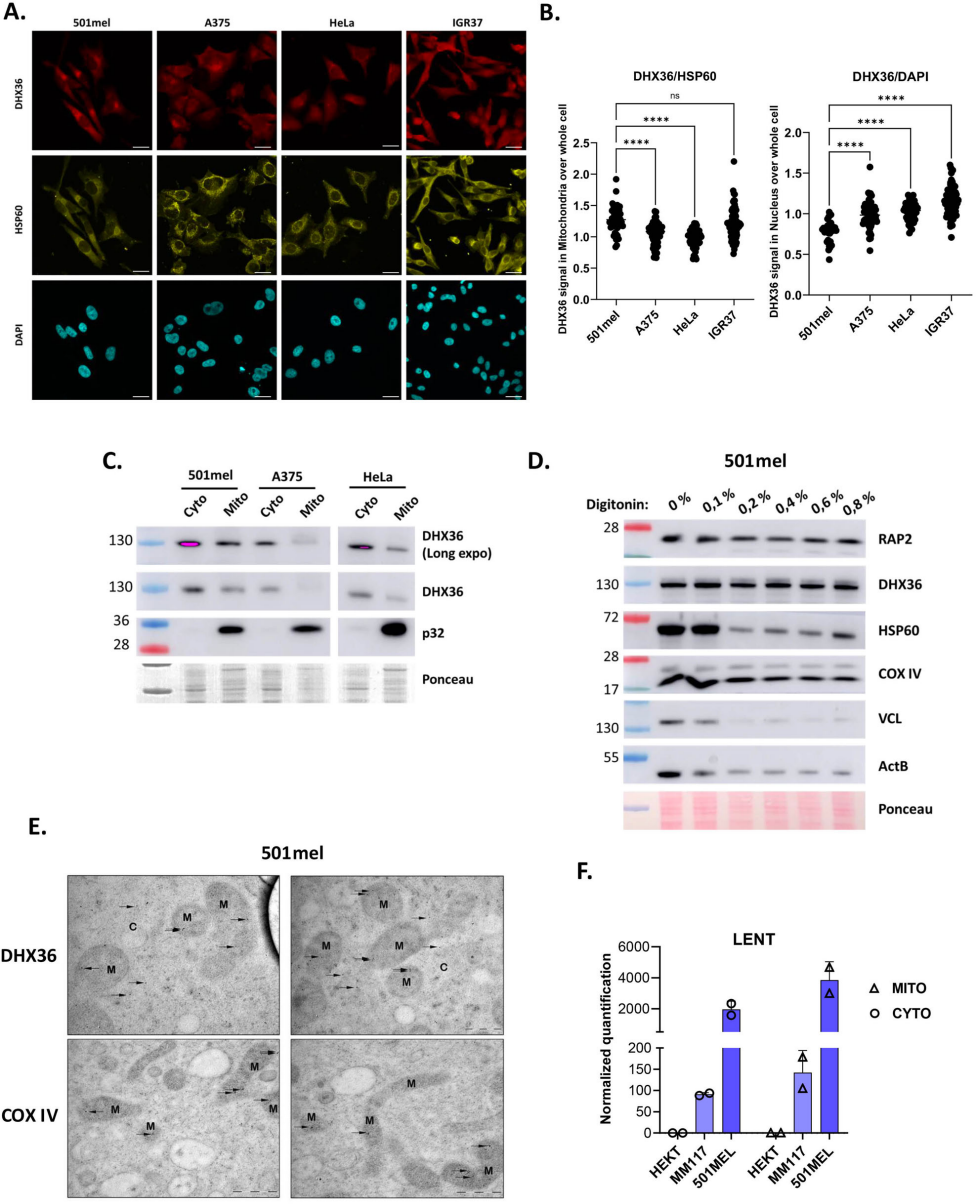
LENT and DHX36 are mainly localized in mitochondria in melanoma cells. **A** Immunofluorescence by confocal microscopy showing DHX36 localization in different cell lines. HSP60 is used as a mitochondria marker and DAPI to stain the nucleus. Scale bars = 10 µM. **B** DHX36 signal was quantified in the whole cell and divided by the signal in mitochondria or in the nucleus. Each measured cell is represented by one point, and groups are compared by one-way ANOVA (Dunnett test). *P < 0.033; **P < 0.0021; ***P < 0.0002; ****P < 0.0001. **C** Immunoblot of cytosolic or mitochondrial fractions of different cell lines. **D** Purified mitochondria were digested with increasing concentrations of digitonin, and retained proteins were analyzed by western blot. **E** Immunogold staining coupled with electron microscopy. Representative stained particles for DHX36 or COX IV in the cytoplasm (C) or mitochondria (M) are indicated with arrows. Scale bars are indicated on images. **F** LENT levels quantified by RT-qPCR in cytosolic or mitochondrial fractions of different cell lines.

Previous studies showed that DHX36 was predominantly cytoplasmic. Immunofluorescence revealed that DHX36 was enriched at cytoplasmic structures in 501Mel, IGR37 and A375 melanoma cells (A375; NCSC-type cells, not expressing LENT) that co-staining with HSP60 identified as mitochondria (Fig. 6A, B). Co-staining with HSP60 was less prominent in HeLa cells, and while little nuclear staining was seen in 501Mel cells, stronger staining was seen in IGR37. ASO-mediated LENT silencing did not modify DHX36 mitochondrial localization, nor did it affect expression of DHX36 mRNA or protein (Fig. S11A–C). Thus, while DHX36 was rather specific to the mitochondria in 501Mel cells, it was more broadly localized to the cytoplasm and even the nucleus in the other lines. Nevertheless, in all tested lines, at least a fraction of DHX36 was found in the mitochondria.

DHX36 association with mitochondria in 501Mel cells was confirmed by immunoblots of cytoplasmic and mitochondrial fractions (Fig. 6C). We then performed immunoblots of the mitochondrial fraction in the presence of increasing quantities of digitonin, which was previously used to assess the association of proteins with mitochondria [32]. While the control Vinculin (VCL) and Beta-actin (ACTB) proteins were rapidly depleted with increasing digitonin concentration, the mitochondrial protein COX IV was resistant to the highest concentrations (Fig. 6D). Both DHX36 and the LENOX-interacting mitochondrial partner RAP2 were also resistant to digitonin, showing they were strongly associated with mitochondria. Consistent with this, COX IV and DHX36 showed resistance to tryptic digestion in swelling buffer, whereas VCL and ACTB were sensitive (Fig. S12A). To consolidate the idea that DHX36 was located in the mitochondria, we performed immune-gold staining coupled to electron microscopy (EM). Numerous DHX36 particles were detected both in the cytoplasm and within the mitochondria, whereas staining for the COX IV displayed as expected mitochondrial localization (Fig. 6E). Hence, in melanocytic melanoma cells, a fraction of DHX36 was localized within the mitochondria. RT-qPCR showed that LENT was also abundant in the mitochondrial fraction from melanoma cells, although we could not discern whether it was within the mitochondria or associated with the outer mitochondrial membrane (Fig. 6F).

Not only was DHX36 associated with mitochondria, but several of the proteins whose translation it regulated were also associated with mitochondria. Immunostaining showed that UBE4A was strongly enriched at mitochondria in 501Mel and IGR37 cells (Fig. S12B). Similarly, while a fraction of NOX4 was present in the nucleus, it was also enriched at mitochondria (Fig. S12C). The transcriptional regulator, RBPJ, was mainly nuclear, but a subfraction was also detected at mitochondria (Fig. S12D). NOX4, RBPJ and UBE4A were further detected by immunoblot in biochemically purified mitochondria (Fig. S12E). These observations supported the idea that a subset of the proteins regulated by the LENT-DHX36 axis was associated with the mitochondria.

### LENT silencing induces autophagy/mitophagy and proteotoxic stress

**Fig. 7:**
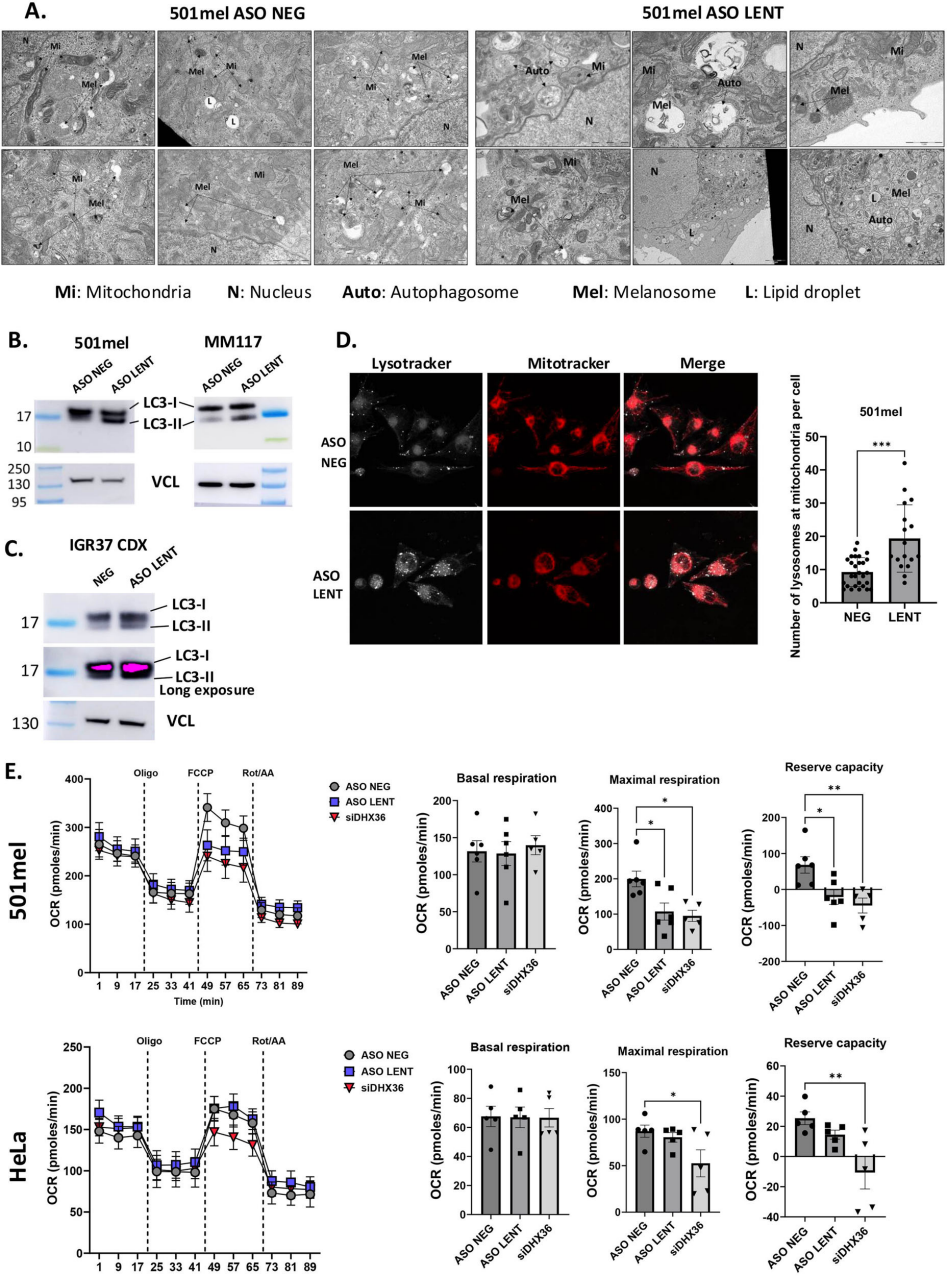
LENT depletion induces mitophagy and reduces oxygen consumption rate. **A** Transmission electron microscopy of 501Mel cells 48 h following transfection of control or LENT-targeting ASO. Autophagosomes and degraded melanosomes were observed in LENT-silenced cells. Scale bars are indicated on the images. **B** Quantification of cytosolic LC3B (LC3-I) and lipid-associated LC3B (LC3 II) by western blot in 501Mel or MM117 cells. Vinculin is used as a loading control. **C** LC3B immunoblot in extracts of IGR37 CDX tumors. **D** Confocal microscopy of unfixed 501Mel cells stained with lysotracker and mitotracker in control or LENT-silenced conditions. The numbers of co-localizing lysosomes and mitochondria was determined and compared between the two conditions by Welch’s test. Scale bars = 10 µM. **E** Oxygen consumption rate was determined upon LENT or DHX36 silencing. Reserve capacity was obtained by subtracting the maximal capacity with the basal capacity. Comparisons were done by one-way ANOVA (Dunnett test). *P < 0.033; **P < 0.0021; ***P < 0.0002; ****P < 0.0001.

The above observations showed that LENT modulated the interaction of mRNAs involved in ER homeostasis, lysosome and autophagy/mitophagy with DHX36 and/or the LP fractions and their subsequent translation. We therefore investigated whether LENT silencing impacted these processes. EM showed that LENT-silenced cells were characterized by lower numbers of mitochondria, but an accumulation of numerous autophagosomes not seen in the control shRNA cells (Auto in Fig. 7A and S13A). Many autophagosomes comprised mitochondria, identifiable by their cristae, indicating extensive mitophagy. In agreement with this observation, immunoblot with anti-LC3 antibody revealed accumulation of the LC3-II form indicative of autophagy in LENT-silenced 501Mel and MM117 cells (Fig. 7B). A more modest but detectable LC3-II accumulation was also observed in extracts from LENT ASO-treated IG37 tumors (Fig. 7C). Staining of control and LENT-silenced cells with both lysotracker and mitotracker showed increased numbers of lysosome-mitochondrial contacts in LENT-silenced cells that was further indicative of auto/mitophagy (Fig. 7D). Accumulation of autophagosomes was not seen in LENOX silenced cells despite that fact that its silencing impacted mitochondrial homeostasis (Fig. S13B) [15]. Thus, induction of autophagy/mitophagy was a major phenotype of LENT silencing.

Given these observations, we asked if mitochondrial function was impacted by profiling the Oxygen Consumption Rate (OCR) using the Agilent SeaHorse. Compared to control ASO, LENT silencing reduced maximal OCR and reserve capacity, but not basal levels in 501Mel cells, whereas no effect was seen in HeLa cells (Fig. 7E). DHX36 silencing reduced maximal and reserve capacities in both cell types revealing its more general role in regulating mitochondrial activity (Fig. 7E). The mitophagy and impaired mitochondrial function upon LENT silencing led to increased ROS levels and the appearance of ROS-high apoptotic cells (Fig. S14A). LENT silencing was further associated with activation of the DNA damage response, with increased gH2AX seen both by immunofluorescence (Fig. S14B) and immunoblot (Fig. S14C). Immunoblot analyses showed close to maximal LC3-II accumulation already 16 h after LENT silencing, whereas gH2AX appeared only after 24 h, suggesting it was a secondary effect of mitophagy (Fig. S14D).

**Fig. 8:**
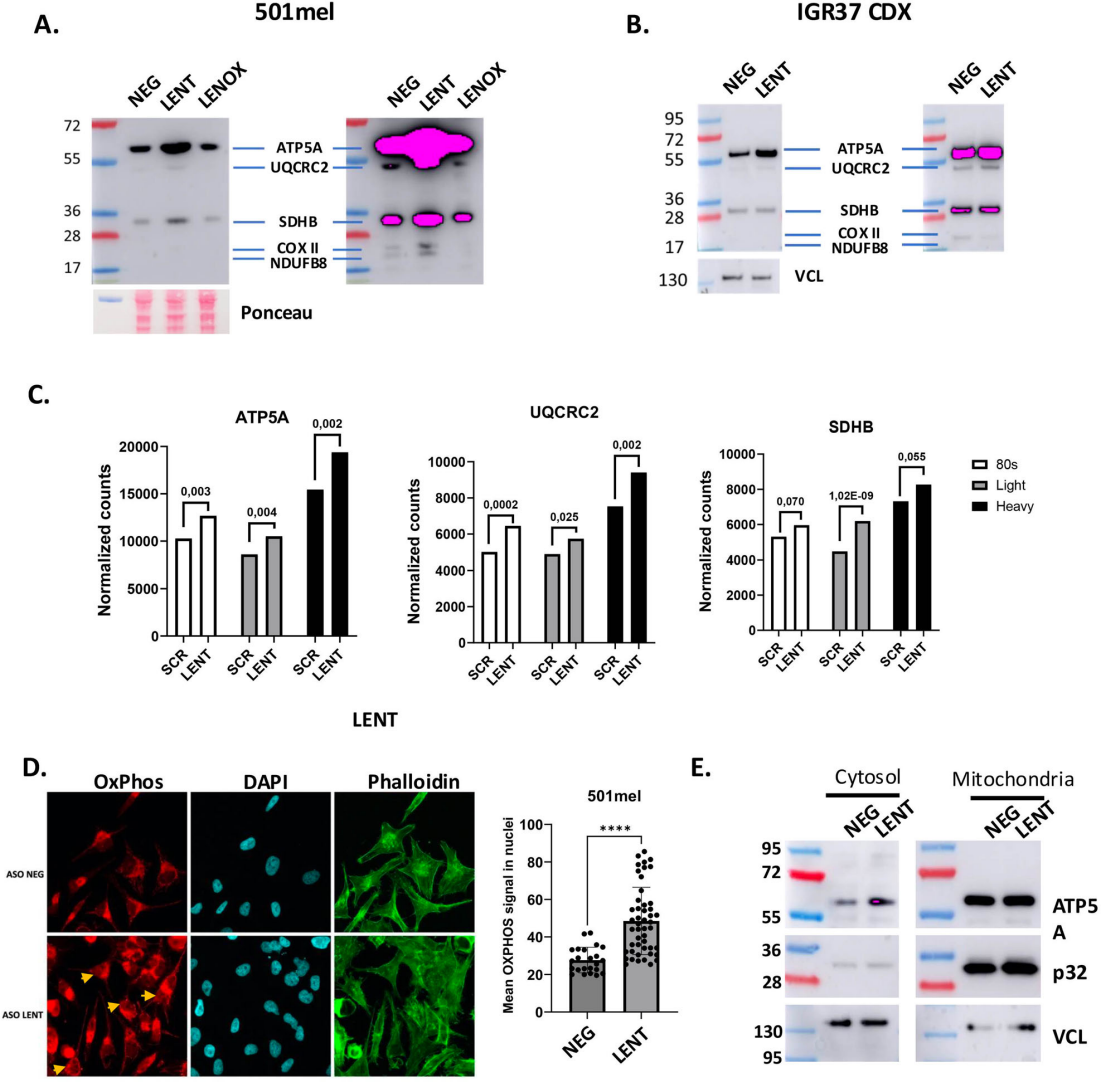
LENT depletion triggers DNA damage and accumulation of OxPhos proteins. **A** Immunoblots detecting the indicated proteins in control or LENT-depleted cells. Panels show different exposures of the same immuoblot. **B** Immunoblots detecting mitochondrial electron transport chain proteins in extracts from IGR-37 CDX tumors. **C** RNA-seq data showing the representation of the indicated RNAs in the 80S, LP and HP fractions. The normalized number of reads is shown along with the adjusted *P*-value between the control and shLENT conditions. **D** OxPhos proteins were detected by confocal microscopy immunofluorescence in control or LENT-silenced cells using phalloidin to stain the cytoplasm. Proteins were quantified in the nucleus using the DAPI signal, and comparisons were made by Mann–Whitney test. Scale bars = 10 µM. Representative nuclei displaying strong OxPhos protein staining are indicated by arrows. **E** Immunoblot of ATP5A1 in cytosolic or mitochondrial fractions upon LENT depletion. Ponceau is shown as loading control. *P < 0.033; **P < 0.0021; ***P < 0.0002; ****P < 0.0001.

The observed autophagy/mitophagy and impaired OxPhos prompted us to investigate changes in the levels of mitochondrial proteins of the electron transport complexes. Strikingly, increased protein levels of ATP5A, UQCR2, SDHB, COX II and NDUFB8 were seen after ASO-mediated LENT silencing in 501Mel cells and in melanocytic Mel888 and MM117 cells (Figs. 8A, S15). In contrast, this accumulation was not seen in LENOX-silenced cells despite the fact that its silencing was also associated with lowered OxPhos capacity [15] (Fig. 8A). Both mitophagy and OxPhos protein accumulation were therefore specific to LENT-silenced cells. Increased ATP5A, the most strongly affected in cells, was also seen in extracts from LENT ASO-treated IGR37 xenograft tumors (Fig. 8B).

OxPhos protein accumulation may represent a compensatory response to the mitophagy and impaired mitochondrial function and/or may at least in part result from their increased translation as the presence of the corresponding mRNAs was upregulated in the polysome fractions from shLENT-silenced cells (Fig. 8C). Furthermore, their accumulation was surprising given the lowered OxPhos, suggesting the excess OxPhos proteins were not mitochondrial, but accumulated in the cytoplasm. Immunofluorescence showed accumulation of mitochondrial proteins at the mitochondria, but also in the cytoplasm and the nucleus of the LENT-silenced cells (Fig. 8D). Immunoblots on the cytosolic and mitochondrial fractions showed accumulation of ATP5A in the cytosolic fraction, with little change in the mitochondrial fraction confirming the immunofluorescence results (Fig. 8E). Nevertheless, the cytoplasmic and mitochondrial ATP5A displayed the same electrophoretic mobility suggesting it represents protein released from mitochondria rather than increased translation of the precursor peptide.

Together, these data are consistent with the idea that LENT silencing impaired ER homeostasis and or lysosome function, resulting in auto/mitophagy with subsequent impaired mitochondrial function. Accumulation of mitochondrial proteins in the cytoplasm and the nucleus may then induce a general proteotoxic stress leading to apoptosis [33–35].

## Discussion

### LENT, a multifunctional lncRNA

Here we characterize LENT as a cytoplasmic lncRNA that interacts with the G4 resolvase DHX36 to promote translation of mRNAs involved in ER homeostasis, lysosomal and mitochondrial function and suppressing autophagy. LENT was primarily expressed in melanocytic, but not mesenchymal-type melanoma cells, and its expression did not correlate with poor survival in primary melanoma, but with poor survival in metastatic melanoma, where it was expressed in proliferative melanocytic type cells marked by an OxPhos signature [15, 36, 37]. ASO-mediated LENT silencing induced apoptosis in cultured melanoma cells and impaired xenograft tumor growth, but did not impact the viability of HEKT cells nor mesenchymal MM047 melanoma cells, where LENT was not expressed, highlighting the specificity of ASO targeting. While the LENT ASO did not show toxicity in mice during the course of the CDX experiment, future development of ASO-based therapy, possibly involving conjugation to nanobodies presenting antibodies against tumor-expressed proteins like MUC1c [38] to enhance tumor-selective delivery, may consolidate ASO-targeting as a candidate therapeutic approach. Moreover, simultaneous ASO targeting of LENT together with LENOX or SAMMSON cooperatively impacted melanoma cell viability. Thus, ASO-targeting of these lncRNAs individually or in combination further highlights their potential as therapeutic targets.

LINC00520 has been the focus of previous studies designated as LASSIE [39] or LEENE [40]. LASSIE was described as an lncRNA induced in endothelial cells by sheer stress that interacts with PECAM-1 to regulate vascular homeostasis by stabilizing adherens junctions. On the other hand, Miao et al. [40] reported that LEENE was induced by pulsatile or oscillatory sheer stress in endothelial cells, but was localized in the nucleus and acted as an enhancer (e)RNA to regulate eNOS expression. In these non-melanoma cells, LINC00520 expression is driven by KLF2 and KLF4, highlighting that transcription factors other than MITF can drive its expression. Moreover, LEENE was further shown to promote transcription of pro-angiogenic genes, angiogenesis and tissue repair following ischemia [41]. In contrast, in melanoma cells, RNA-scope and cell fractionation showed that LENT was distributed between the cytosol and the mitochondria. We did not see enrichment of PECAM-1 in the RNA pulldown/mass spectrometry experiments, and LENT silencing did not affect NOS3 (eNOS) expression. This comparison between our data and that previously reported shows that LINC00520 is a multifunctional lncRNA [42] functioning in a cell-type and context-dependent manner as an eRNA in the nucleus to regulate gene expression or in the cytoplasm to regulate translation.

### LENT coordinates the ribosomal association of mRNAs encoding proteins involved in ER and mitochondrial homeostasis in melanocytic melanoma cells

We found that LENT selectively and directly interacted with the DHX36 G4 resolvase, suggesting that it may contain a G4 structure. The in vitro interaction between LENT and DHX36 was reduced, but not abolished by mutation of a G-rich sequence with a low predicted potential to form a G4 structure. Nevertheless, this G-rich sequence diverges from more canonical G4-forming sequences and LENT does not comprise the 5’-GGnGGnGG-3’ motif or other motifs previously shown to be enriched in DHX36-associated RNAs. G4-forming sequences are, however, variable with the length and sequence of the loop regions between the G blocks contributing to selectivity [43, 44] with DHX36 showing high specificity for parallel G4 structures [22]. Moreover, LENT specifically pulled down DHX36, but no other well-characterized G4 resolvases, such as the RECQL-family, nor DDX5, DDX11, DHX9 or DDX3×45 [28, 44, 45]. DDX3X, DDX5 and DHX9 are reported RNA G4 resolvases [28, 45, 46]. The basis for the selectivity of the LENT-DHX36 interaction and experimental confirmation of a G4 structure in LENT remain to be determined.

We provide evidence that LENT modulates DHX36 interaction with a subset of mRNAs. DHX36-RIP from control 501Mel cells identified DHX36-associated RNAs. The most strongly associated RNAs were enriched in potential G4-forming motifs and the 5’-GGnGGnGG-3’ motif. DHX36 RIP upon LENT silencing identified RNAs whose association with DHX36 was either increased or decreased. Increased binding may be explained if LENT were to comprise a G4 structure that simply competes with the G4s in other RNAs for DHX36 binding. However, this competition mechanism cannot explain the reduced binding of RNAs with DHX36 seen upon LENT silencing. How LENT binding to DHX36 modifies its interaction with these RNAs in a positive or negative manner remains to be determined.

An important observation of this study is the association of DHX36 with ribosomes, in the 80S and the polysome fractions, as previously reported [45]. The presence of LENT in the 80S and light polysome fractions suggested either that it was itself translated (LENT comprises several short ORFs) and/or that it may modulate the selectivity of DHX36 to resolve G4 structures in target mRNAs promoting/inhibiting their translation. In accordance with this idea, LENT silencing resulted in a selective depletion of a set of mRNAs in the LP fractions. Association of many of these mRNAs with the 80S and HP fractions was also reduced, but to a lesser extent. Of these, 74 showed reduced interaction with DHX36 upon LENT silencing. In contrast, several mRNAs whose association with DHX36 was upregulated upon LENT silencing were enriched in the 80S, LP and HP fractions. Immunoblots showed that mRNAs whose engagement with the LP fractions was promoted by LENT were less well translated upon its silencing and vice versa.

Together, the above results support the idea that LENT, via interaction with DHX36, positively or negatively regulates the engagement of mRNAs with polysomes and fine-tunes their subsequent translation. However, while there was a strongly significant overlap, not all transcripts showing reduced association with the LP fraction displayed reduced interaction with DHX36 upon LENT silencing. It is possible that their interactions with DHX36 were less stable or that DHX36 only acts during the elongation steps of their translation, once they are engaged with the ribosome. Alternatively, we cannot formally exclude the existence of alternative DHX36-independent mechanisms by which LENT regulates its polysome engagement and translation. Despite the above caveats, the idea that the LENT-DHX36 axis regulates translation is in accordance with previous studies reporting that DHX36 unwinds G4 structures in mRNA to regulate their association with ribosomes and their translation in HEK293T, HeLa and muscle stem cells [45, 47].

LENT is not the first lncRNA shown to affect DHX36 activity. Matsumura et al. [48] identified a cytoplasmic G4-containing lncRNA designed GSEC that binds and inhibits DHX36 promoting motility of colon cancer cells. Similarly, SMaRT is an lncRNA that binds the G4 of the MLX-γ isoform, preventing unwinding by DHX36 and repressing its translation in murine muscle differentiation [49]. Nevertheless, while GSEC and SMaRT seem to act as molecular decoys to inhibit DHX36 function, LENT is unique in its ability to both positively and negatively impact DHX36 function in an RNA-selective manner.

### LENT suppresses autophagy to promote melanoma cell survival

LENT promotes engagement of a collection of mRNAs with the LP fractions. Strikingly, these mRNAs are strongly enriched in multiple aspects of ER and protein homeostasis encoding numerous subunits of several protein complexes or pathways, such as SEL1-SYNV1 required for ERAD, TAP1, TAP2 and CALR involved in HLA transport and antigen presentation or enzymes and machinery involved protein glycosylation. It was recently reported that translation of proteins involved in ER and secretome function is enriched at ER-associated ribosomes, while that of many mitochondrial proteins is enriched at the OMM [50]. Despite the observation that LENT and DHX36 were enriched at the mitochondria, the selective depletion of these mRNAs associated with ER function suggests that LENT was rather regulating the engagement of a subset of mRNAs at ER-associated ribosomes. Reduced translation of these mRNAs would be expected to lead to accumulation of misfolded and/or mis-localized proteins. For example, downregulation of WFS1 has previously been shown to lead to reduced OxPhos capacity, increased mitochondrial-lysosome contact and mitophagy similar to what was observed here [51, 52]. Consequently, a major phenotype of LENT silencing is autophagy and mitophagy that rapidly appeared in LENT-silenced cells associated with impaired OxPhos capacity.

Accompanying the autophagy/mitophagy is the accumulation of mitochondrial proteins in the cytoplasm and nucleus. As the cytoplasmic accumulated ATP5A had the same electrophoretic mobility as the mitochondrial fraction, it was possible that it represents protein released from mitochondria rather than via increased translation and/or impaired ER-degradation of the precursor peptide. We note that DHX36 was detected within the mitochondria and hence has the potential to regulate translation of both nuclear and mitochondrial encoded transcripts, although this will require further investigation. There is, however, no evidence that LENT itself is located within the mitochondria, as it was not detected among the transcripts enriched in mitochondria-targeted APEX-seq experiments in melanocytic melanoma cells [53].

Notwithstanding the source of the mitochondrial protein accumulation, it preceded activation of the DNA damage response, suggesting that it elicited proteotoxic stress, leading finally to apoptosis. We also note that LENT silencing may impact cholesterol metabolism, with reduced cholesterol levels known to promote autophagy. The mRNA encoding HMGCR, the rate-limiting enzyme in cholesterol synthesis, was among those depleted in the LP fraction. Similarly, the mRNA encoding DHCR7, which catalyzes the final step of cholesterol synthesis, was among the 74 common transcripts reduced in interaction with DHX36 and the LP fraction. Our data therefore support the idea that the major function of LENT is to fine-tune translation and optimize multiple cellular functions that maintain OxPhos capacity, suppress autophagy/mitophagy and promote survival and proliferation of melanocytic melanoma cells.

The results reported here show how the LENT-DHX36 axis may regulate mitochondrial homeostasis and OxPhos capacity through suppression of mitophagy. Previous studies showed that lncRNA SAMMSON interacted with XRN2, CARF and the mitochondrial protein p32 to coordinate cytoplasmic and mitochondrial translation in melanoma cells [13, 14]. Additionally, we previously reported how LENOX regulated mitochondrial fission/fusion and OxPhos capacity through interaction with the RAP2C and DRP1 GTPases [15]. Moreover, melanoma cells also express the mitochondria-located lncRNA ROSALIND that acts to protect ribosomal proteins from oxidation and optimize mitochondrial function [53]. These results highlight how these lncRNAs interact with different effector proteins to regulate diverse pathways, with, as a common outcome, optimization of mitochondrial function and OxPhos capacity [54].

Why melanocytic melanoma cells, as opposed to mesenchymal melanoma cells, where LENT is not expressed, specifically require fine-tuning of translation and high OxPhos capacity is unclear. However, one likely possibility is that melanocytic melanoma cells, like normal melanocytes, often synthesize melanin, a process that involves the production of reactive oxygen species, rendering them particularly vulnerable to oxidative stress [55, 56]. Optimization of mitochondrial and ER function may therefore be essential to antagonize oxidative stress and may therefore explain why proliferative melanoma cells exploit such diverse mechanisms to optimize mitochondrial homeostasis and OxPhos capacity and ensure cell viability.

## Supplementary information

Supplemental Figures and Legends

Complete Immunoblots

Supplemental dataset 1

Supplemental dataset 2

Supplemental dataset 3

Supplemental dataset 4

Supplemental dataset 5

aj-Checklist
