# Supplementary figures and images for: Interaction of lncRNA LENT with DHX36 regulates translation and suppresses autophagy in melanoma
Source: Cell Death Dis. 2025 Dec 19;17(1):121. doi: 10.1038/s41419-025-08296-3 (PMC12847983; doi:10.1038/s41419-025-08296-3)

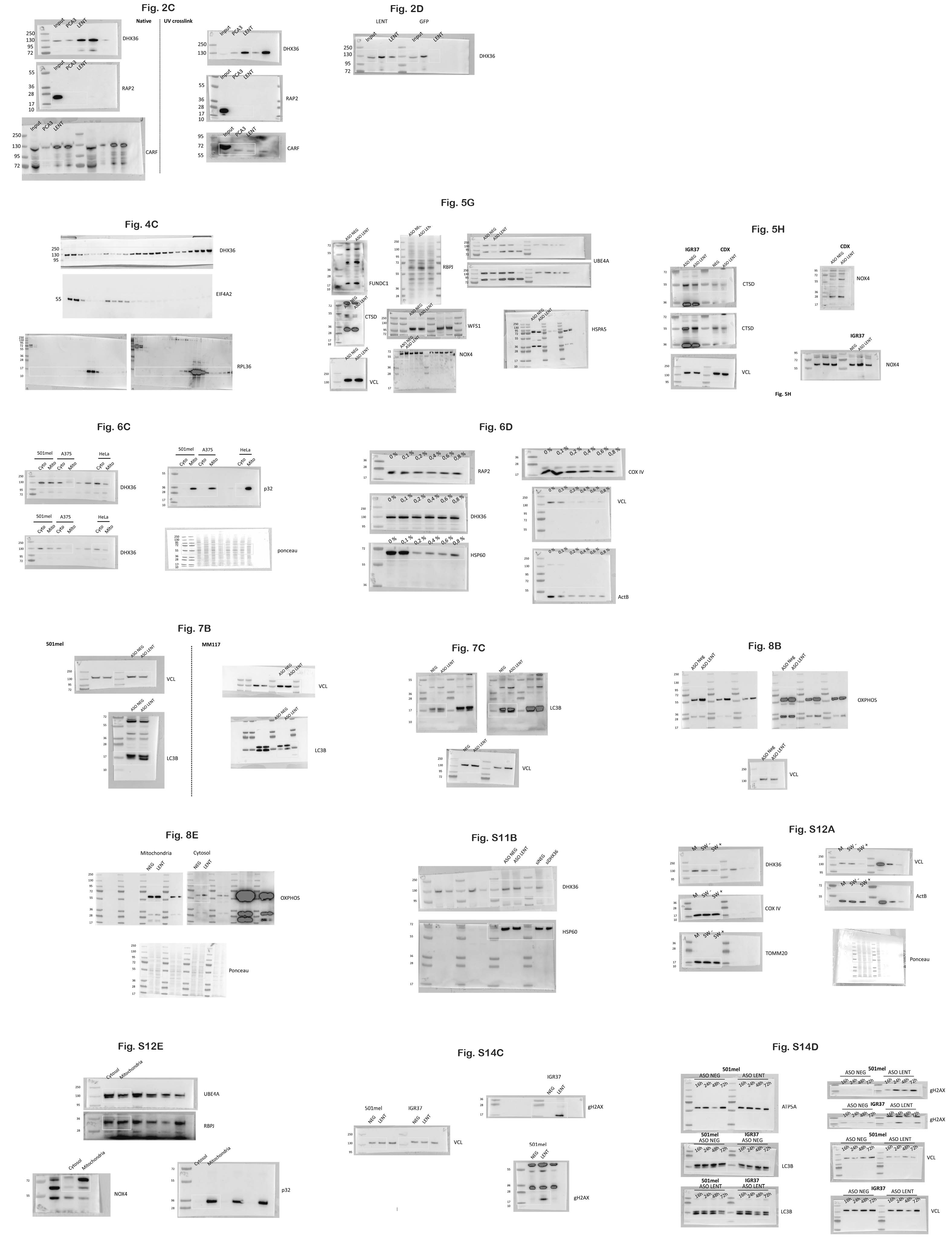

Supplement: Supplementary file 2 — Complete Immunoblots [file 41419_2025_8296_MOESM2_ESM.jpg]
