# Supplementary material for: Interaction of lncRNA LENT with DHX36 regulates translation and suppresses autophagy in melanoma
Source: Cell Death Dis. 2025 Dec 19;17(1):121. doi: 10.1038/s41419-025-08296-3 (PMC12847983; doi:10.1038/s41419-025-08296-3)
Supplement: Supplementary file 8 — aj-Checklist [file 41419_2025_8296_MOESM8_ESM.pdf]

SPRINGER NATURE

Corresponding Author Name: DAVIDSON Irw
Manuscript Number: 25-1894-T

Reporting Summary

Springer Nature wishes to improve the reproducibility of the work that we publish. This checklist is used to ensure good reporting standards and to improve the reproducibility. Please respond completely to all questions relevant to your manuscript. For more information, please read the journal's Guide to Authors.

☑ **Check here to confirm that the following information is available in the Material & Methods section:**

- **The exact sample size (*n*)** for each experimental group/condition, given as a number, not a range
- **A description of the sample collection** allowing the reader to understand whether the samples represent technical or biological replicates (including how many animals, litters, culture, etc.)
- **A statement of how many times the experiment shown was replicated in the laboratory**
- **Definitions of statistical methods and measures**: For small sample sizes (n<5) descriptive statistics are not appropriate, instead plot individual data points
  - Very common tests, such as *t*-test, simple χ^2 tests, Wilcoxon and Mann-Whitney tests, can be unambiguously identified by name only, but more complex techniques should be described in the methods section
  - Are tests one-sided or two-sided?
  - Are there adjustments for multiple comparisons?
  - **Statistical test results**, e.g., ***P* values**
  - Definition of **'center values'** as **median or mean**;
  - Definition of **error bars as s.d. or s.e.m. or c.i.**

Please ensure that the answers to the following questions are reported in the manuscript itself. We encourage you to include a specific subsection in the methods section for statistics, reagents and animal models. Below, provide the page number or section and paragraph number.

Statistics and general methods

	Reported in section/paragraph or page #
1. How was the sample size chosen to ensure adequate power to detect a pre-specified effect size? (Give section/paragraph or page #)	Materials and Methods, lines 387-388.
For animal studies, include a statement about sample size estimate even if no statistical methods were used.	Materials and Methods, line 389.
2. Describe inclusion/exclusion criteria if samples or animals were excluded from the analysis. Were the criteria pre-established? (Give section/paragraph or page #)	Materials and Methods, line 390.
3. If a method of randomization was used to determine how samples/animals were allocated to experimental groups and processed, describe it. (Give section/paragraph or page #)	N/A. No specific methods for randomization was used.
For animal studies, include a statement about randomization even if no randomization was used.	Materials and Methods, line 212.

4. If the investigator was blinded to the group allocation during the experiment and/or when assessing the outcome, state the extent of blinding. (Give section/paragraph or page #)	N/A. No blinding was performed
For animal studies, include a statement about blinding even if no blinding was done.	Materials and Methods, line 216.
5. For every figure, are statistical tests justified as appropriate?	Material and Methods line 387-388
Do the data meet the assumptions of the tests (e.g., normal distribution)?	Materials and Methods, line 389.
Is there an estimate of variation within each group of data?	Materials and Methods, line 392.
Is the variance similar between the groups that are being statistically compared? (Give section/paragraph or page #)	Materials and Methods, line 391-392.

Reagents

Reported in section/paragraph or page #

6. Report the source of antibodies (vendor and catalog number)	Materials and Methods, lines 403-405
7. Identify the source of cell lines and report if they were recently authenticated (e.g., by STR profiling) and tested for mycoplasma contamination	Materials and Methods, lines 94-111.

Animal Models

Reported in section/paragraph or page #

8. Report species, strain, sex and age of animals	Materials and Methods, lines 202-216.
9. For experiments involving live vertebrates, include a statement of compliance with ethical regulations and identify the committee(s) approving the experiments.	Materials and Methods, lines 202-208

10. We recommend consulting the ARRIVE guidelines (*PLoS Biol.* **8**(6), e1000412,2010) to ensure that other relevant aspects of animal studies are adequately reported.

Human subjects

Reported in section/paragraph or page #

Item	Reported in section/paragraph or page #
11. Identify the committee(s) approving the study protocol.	N/A No human subjects
12. Include a statement confirming that informed consent was obtained from all subjects.	N/A No human subjects
13. For publication of patient photos, include a statement confirming that consent to publish was obtained.	N/A No human subjects
14. Report the clinical trial registration number (at ClinicalTrials.gov or equivalent).	N/A No human subjects

15. For phase II and III randomized controlled trials, please refer to the CONSORT statement and submit the CONSORT checklist with your submission.

16. For tumor marker prognostic studies, we recommend that you follow the REMARK reporting guidelines.

Data deposition

Reported in section/paragraph or page #

Item	Reported in section/paragraph or page #
17. Provide accession codes for deposited data. Data deposition in a public repository is mandatory for:<br>a. Protein, DNA and RNA sequences<br>b. Macromolecular structures<br>c. Crystallographic data for small molecules<br>d. Microarray data	Data availability statement: Materials and Methods lines 396-398

Deposition is strongly recommended for many other datasets for which structured public repositories exist; more details on our data policy are available in the Guide to Authors. We encourage the provision of other source data in supplementary information or in unstructured repositories such as Figshare and Dryad. We encourage publication of Data Descriptors (see Scientific Data) to maximize data reuse.

Item	Reported in section/paragraph or page #
18. If computer code was used to generate results that are central to the paper's conclusions, include a statement in the Methods section under "**Code availability**" to indicate whether and how the code can be accessed. Include version information as necessary and any restrictions on availability.	N/A no custom computer code was used, only standard publically available softwares
